# A semiconductor-electrocatalyst nano interface constructed for successive photoelectrochemical water oxidation

**DOI:** 10.1038/s41467-023-38285-z

**Published:** 2023-05-04

**Authors:** Zilong Wu, Xiangyu Liu, Haijing Li, Zhiyi Sun, Maosheng Cao, Zezhou Li, Chaohe Fang, Jihan Zhou, Chuanbao Cao, Juncai Dong, Shenlong Zhao, Zhuo Chen

**Affiliations:** 1 Energy & Catalysis Center, Department of Materials Physics and Chemistry, School of Materials Science and Engineering, Beijing Institute of Technology, Beijing, 100081 China; 2 Beijing Synchrotron Radiation Facility, Institute of High Energy Physics, Chinese Academy of Sciences, Beijing, China; 3 College of Chemistry and Molecular Engineering, Beijing National Laboratory for Molecular Sciences, Peking University, Beijing, China; 4 CNPC Research Institute of Petroleum Exploration & Development, Beijing, 100083 China; 5 School of Chemical and Biomolecular Engineering, The University of Sydney, Sydney, NSW Australia

**Keywords:** Electrocatalysis, Artificial photosynthesis, Photocatalysis

## Abstract

Photoelectrochemical water splitting has long been considered an ideal approach to producing green hydrogen by utilizing solar energy. However, the limited photocurrents and large overpotentials of the anodes seriously impede large-scale application of this technology. Here, we use an interfacial engineering strategy to construct a nanostructural photoelectrochemical catalyst by incorporating a semiconductor CdS/CdSe-MoS_2_ and NiFe layered double hydroxide for the oxygen evolution reaction. Impressively, the as-prepared photoelectrode requires an low potential of 1.001 V vs. reversible hydrogen electrode for a photocurrent density of 10 mA cm^−2^, and this is 228 mV lower than the theoretical water splitting potential (1.229 vs. reversible hydrogen electrode). Additionally, the generated current density (15 mA cm^−2^) of the photoelectrode at a given overpotential of 0.2 V remains at 95% after long-term testing (100 h). *Operando* X-ray absorption spectroscopy revealed that the formation of highly oxidized Ni species under illumination provides large photocurrent gains. This finding opens an avenue for designing high-efficiency photoelectrochemical catalysts for successive water splitting.

## Abstract (web summary)

The limited photocurrents and large overpotentials impede practical application of photoelectrochemical water splitting. Here, the authors construct a CdS-CdSe/MoS2/NiFe layered double hydroxides photoanode, delivering a low potential and large photocurrent gains due to the formation of highly oxidized Ni species under illumination

## Introduction

Acquiring clean hydrogen from water is a fascinating approach with which to cope with the increased energy demand and the environmental challenges^1^. In recent decades, substantial effort has been expended to develop efficient and stable photo/electrochemical (PEC) water-splitting systems^2–7^. Generally, electrolysers have the advantages of scalability, large production and high stability under various circumstances. However, the large overpotentials of the electrodes greatly increase the energy consumed by the hydrogen production process^8–10^. In contrast, photochemical technologies are more energy-saving and potentially cost-effective, but their intermittent nature and low energy conversion efficiencies seriously impede large-scale application. Therefore, it is highly desirable to develop solar-driven electrochemical technology that combines the advantages of both electrochemical and photochemical processes.

To realize high efficiency and continuity with the abovementioned water splitting system, various catalysts/electrodes have been prepared from a variety of nanomaterials, including metal oxides, hydroxides and carbons. Semiconductors are widely used as photoelectrocatalysts to convert solar energy into electricity and reduce the potential required for water splitting, thereby decreasing the cost of hydrogen production^11^. However, the vast majority of semiconductors are unsuitable for industrial-scale application owing to their limited maximum photocurrents, relatively high costs and susceptibilities to photocorrosion under harsh conditions (e.g., high applied potentials and strongly alkaline or acidic electrolytes)^12^. In contrast, transition metal oxides and carbon-based materials afford high current densities and applied potentials, but their narrow bandgaps and large overpotentials caused by the sluggish kinetics of the oxygen evolution reaction (OER) remain challenges^13,14^.

We suggest that construction of nanoscale interfacial heterojunctions would be an effective strategy for acquiring high-performance PEC catalysts: (1) the nanoscale interfaces could collect electrons to drive the fuel-forming reactions at low overpotentials; (2) nanojunctions create high percentages of effective active surfaces with accelerated electron-hole separation efficiencies to assure high catalytic activity; and (3) a synergistic effect between the semiconductor and catalyst would enable electronic and structural changes in the active species during illumination and stabilize the light absorbers simultaneously, which is key for improving both the activity and stability of the PEC process.

In this work, a CdS-CdSe/MoS_2_/NiFe-LDH (TQ-NiFe) nanoarchitecture is synthesized by using a facile hydrothermal method, and the PEC performance is examined in 1 M KOH solution under 1.5 G illumination. Impressively, the TQ-NiFe sample affords high PEC performance with an onset potential of 0.243 V vs. reversible hydrogen electrode (RHE) and a high photocurrent density of 10.9 mA cm^−2^ at 1.23 V vs. RHE, which is approximately double those of other state-of-the-art photoanodes^15,16^. Furthermore, the current density of the as-prepared anode exhibits a 2.8-fold enhancement in the current density from 42 to 118 mA cm^−2^ at potentials up to 1.65 V once illumination is introduced. More importantly, with a high current density of 108 mA cm^−2^, only 12% decay is observed after 100 h of continuous electrolysis. The active sites and mechanism of the as-fabricated TQ-NiFe during the PEC catalytic process have been clearly elucidated by *operando* X-ray absorption spectroscopy (XAS) experiments.

## Results

### Design principles and characterization

**Fig. 1:**
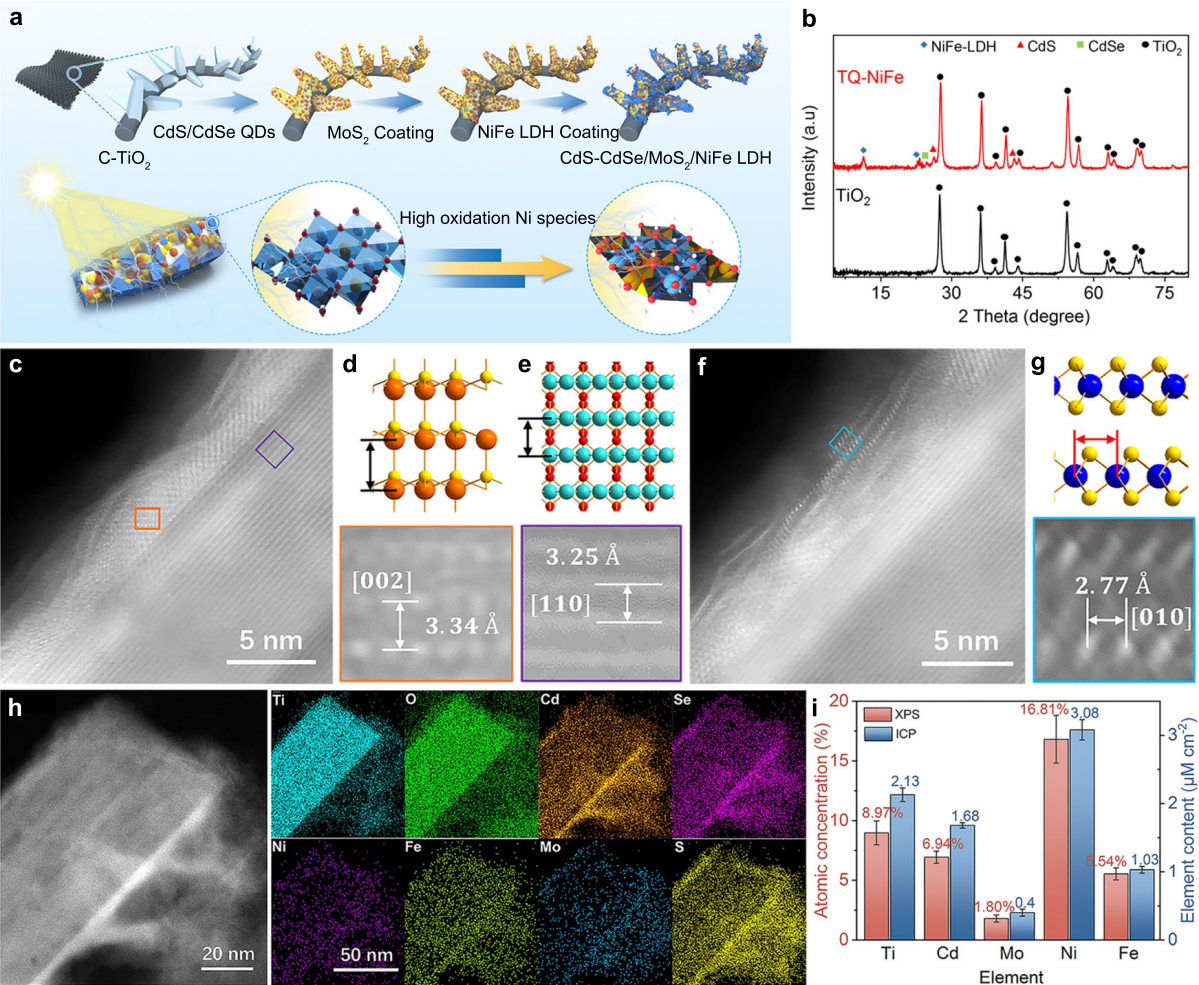
Characterizations of catalysts. **a** Schematic illustration of the TQ-NiFe grown on the carbon cloth. **b** X-ray diffraction patterns for the as-prepared TQ-NiFe and TiO_2_. **c** Atomic resolution HAADF-STEM images of TiO_2_ (lattice in purple frame) and CdS-CdSe (lattice in orange colour). **d** Magnified image of CdS in (**c**) and the corresponding crystal model. **e** Magnified image of TiO_2_ in (**c**) and the corresponding crystal model. **f** HAADF-STEM image of MoS_2_ (lattice in blue colour). **g** Magnified image of MoS_2_ in (**f**) and the corresponding crystal model. **h** EDS maps of the TiO_2_/CdS-CdSe/MoS_2_/NiFe-LDH structure. **i** Quantitative data for the main metallic elements in TQ-NiFe, as determined with X-ray photoelectron spectroscopy and inductively coupled plasma‒mass spectrometry.

Figure 1a shows the synthetic process used to prepare the nanostructured TiO_2_-CdS/CdSe-MoS_2_-NiFe-LDH (TQ-NiFe) photoanodic catalysts. To furnish a larger surface area and shorter diffusion pathways for photogenerated minority carriers, well-aligned one-dimensional (1D) titanium dioxide (TiO_2_) nanoarrays with diameters of ~300 nm (Supplementary Figs. 1 and 2) were first grown on a carbon matrix^7,17–20^. Then, CdS/CdSe quantum dots, MoS_2_ and NiFe layered double hydroxides (LDHs) were deposited in situ on the as-prepared TiO_2_ arrays to serve as the photosensitive layer, protector and catalytic layer, respectively. As reported^21^, the CdS/CdSe quantum dots on the TiO_2_ nanoarrays served as light absorbers to broaden the light absorption range and facilitate charge separation^22^. Molybdenum disulfide was introduced to stabilize the quantum dots on the surfaces of the TiO_2_ nanoarrays^23^. This sandwich-like structure improves the charge separation efficiency and photocorrosion resistance and enhances the catalytic activity via a synergistic effect among the components.

The TQ-NiFe nanocomposite was synthesized by using a two-step hydrothermal process (see the experimental procedures). Scanning electron microscopy (SEM) images (Supplementary Fig. 1b) showed that the morphology of the as-prepared TQ-NiFe showed no obvious change except that the surface was rougher that those of the original TiO_2_ nanoarrays. Furthermore, the chemical composition and structure of the synthesized TQ-NiFe were analyzed with atomic resolution high-angle annular dark field scanning transmission electron microscopy (HAADF-STEM), energy dispersive spectroscopy (EDS), X-ray diffraction (XRD) and X-ray photoelectron spectroscopy (XPS) (Supplementary Figs. 2–5). X-ray diffraction (XRD) measurements (Fig. 1b) were performed to investigate the composition of the as-prepared TQ-NiFe. As shown in Fig. 1b, the typical peaks for CdSe (JCPDS No. 88-2346), CdS (JCPDS No. 75-1546) and the LDHs (JCPDS No. 40-0215) appeared in the XRD pattern for the TQ-NiFe, and peaks for CdSe and CdS had already emerged for the TQ (Supplementary Fig. 6). The atomic resolution HAADF-STEM images and EDS mapping of TQ-NiFe are shown in Fig. 1c–h. The lattice fringe spacings of 0.334 and 0.325 nm seen in the HAADF-STEM images (Fig. 1c–e) of TQ-NiFe corresponded to the (002) planes of CdS and the (110) plane of TiO_2_, respectively. Figure 1c, f shows a continuous coating of the MoS_2_ layer on CdS/Se with a lattice fringe spacing (Fig. 1g) of 0.277 nm, which corresponds to the (010) planes^24,25^. The EDS maps shown for TQ-NiFe in Fig. 1h confirmed the uniform composition of the photosensitive layer, protector and catalytic layer. In addition, to verify the universality of the MoS_2_ coating, more areas were selected, and the corresponding evidence is shown in Supplementary Figs. 5 and 6. The two peaks located at 383.4 cm^−1^ and 408.1 cm^−1^ in the Raman spectrum of TQ verified the presence of MoS_2_ (Supplementary Fig. 7). Furthermore, the HRTEM-EDS (Supplementary Figs. 8 and 9) and XPS analyses (Supplementary Figs. 10 and 11) confirmed that the prepared samples were composed of Cd, Mo, Ni, Fe, Se, S and O elements without other impurities. Quantitative measurements by inductively coupled plasma‒mass spectrometry (ICP‒MS) revealed that TQ-NiFe contained 3.08 μM cm^−2^ Ni, 1.03 μM cm^−2^ Fe, 2.13 μM cm^−2^ Ti, 1.68 μM cm^−2^ Cd and 0.4 μM cm^−2^ Mo (Fig. 1i), and the element proportions were very close to the values calculated based on the XPS data.

### Photoanode photoelectrochemical (PEC) performance

**Fig. 2:**
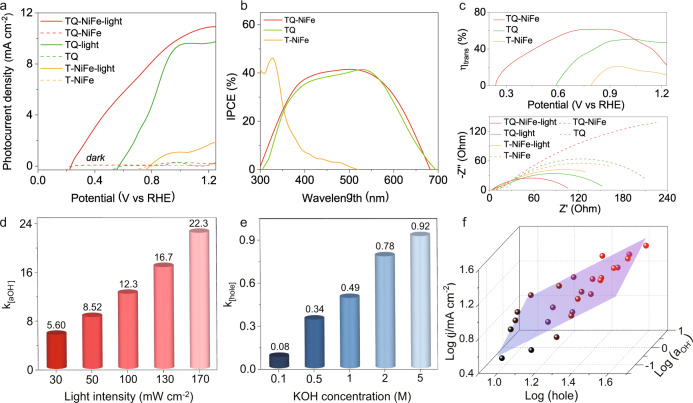
Photoelectrochemical measurements. **a** Photoelectrocatalytic properties of TQ-NiFe, TQ and T-NiFe. **b** IPCE spectra of TQ-NiFe, TQ and T-NiFe collected over the incident light wavelength range 300 to 700 nm at 0.9 V. **c** Charge transfer efficiencies of TQ-NiFe, TQ and T-NiFe; electrochemical impedance spectra of TQ-NiFe, TQ and NiFe. **d** Fitted dynamic reaction rate constants with hydroxide ion activities under different illumination intensities. **e** Fitted dynamic reaction rate constant with hole concentrations for different KOH concentrations. **f** Fitted dynamic surface of the TQ-NiFe.

The PEC performance of TQ-NiFe in the water splitting reaction was evaluated in 1 M KOH (pH = 13.6) with a standard three-electrode system under 1.5 G illumination. As shown in Fig. 2a, an obvious increase in the current density was observed after introducing the light. A sharply increased anodic photocurrent starting at an onset potential (*E*_onset_) of 0.243 V (defined as the potential required to reach an OER photocurrent density of 0.1 mA cm^−2^) appeared for the TQ-NiFe electrode, indicating markedly improved catalytic activity compared to the other electrodes (the *E*_onset_ values for TQ and T-NiFe were 0.588 V and 0.796 V, respectively). In addition to *E*_onset_, the operational potential at 10 mA cm^−2^ (*E*_*j*=10_) is another key to achieving a solar-to-fuel conversion device with 10% efficiency. The TQ-NiFe electrode exhibited a very low potential of 1.001 V at 10 mA cm^−2^, which was considerably smaller than those of TQ (1.34 V) and T-NiFe (1.57 V). Furthermore, the TQ-NiFe electrode also showed improved stability due to the MoS_2_ protective layer, which protected the cadmium chalcogenide from photocorrosion in the solution under illumination and promoted the accumulation of photogenerated holes in the CdSe layer for further transport to the outside layer via a potential gradient (Supplementary Fig. 12)^26–28^. The strong OER performance of the TQ-NiFe electrode was confirmed by comparing it with those of other cadmium sulfide-based photoelectrocatalysts under similar conditions (Supplementary Fig. 13, Supplementary Tables 1 and 2)^29–32^.

To evaluate the photocatalytic properties of the TQ-NiFe, key indicators, including photocarrier generation, transfer/injection of the photocarrier, and the surface reaction rate, were measured. The UV‒vis absorption spectra in Supplementary Fig. 14 show that TQ-NiFe and TQ exhibited absorption edges at approximately 750 nm and wider absorption ranges than T-NiFe (with a light absorption edge at ~480 nm). The broader range of the photoresponse was also confirmed with the incident-photon-to-current conversion efficiency (IPCE) curve. As shown in Fig. 2b, T-NiFe displayed a narrow peak with an edge at approximately 430 nm and a slightly tailing absorption band out to ~480 nm. However, TQ-NiFe exhibited a photocurrent response range of 300–680 nm with a high IPCE of 40% in the visible region. There was a discrepancy between the photocurrent and the integrated IPCE results, which could be attributed to a trap filling effect because the IPCE measurement was conducted with low optical power (Supplementary Fig. 15). The dark current and Mott-Schottky plots of the TQ-NiFe are shown in Supplementary Fig. 16. Butler–Volmer kinetics were used to model electron transfer between the catalyst and the redox couple. The fitting results indicated that the exchange current density for TQ-NiFe was three orders of magnitude higher than that of T-NiFe. Additionally, the carrier concentration of TQ-NiFe was 13 times higher than that of TQ-NiFe. The increased carrier concentration seen after introducing the CdS/CdSe quantum dots caused the Femi level to rise, thereby causing TQ-NiFe to have a larger *V*_oc_ than T-NiFe. Therefore, the larger exchange current density and open-circuit voltage of the TQ-NiFe could be responsible for its larger potential window. Furthermore, the charge transfer efficiency (*η*_trans_) is presented in the photoconversion efficiency plots (Supplementary Fig. 17). The upper panel of Fig. 2c indicates that the maximal charge transfer efficiency calculated for the TQ-NiFe sample was 61.5% (at 0.86 V vs. RHE), which was much higher than those of TQ (50.6% at 1.02 V vs. RHE) and T-NiFe (21.0% at 0.94 V vs. RHE). The decreased optimal potential of the TQ-NiFe sample also confirmed the merits of the reduced charge transfer resistance, which facilitated electron-hole separation and transport. As shown in the electrochemical impedance spectrum (EIS), TQ-NiFe exhibited the largest impedance under dark conditions, which mainly resulted from the higher resistance of the TQ layer (bottom part of Fig. 2c). Under illumination, however, the impedance of TQ-NiFe was significantly reduced and was much smaller than those of T-NiFe and TQ, which confirmed its fast charge transfer capacity and the favorable gradient of the energy level.

Subsequently, the surface reaction kinetics of TQ-NiFe were investigated by determining the appropriate kinetic model for the OER^33–37^. To verify whether the equivalent circuit model (Supplementary Fig. 18) we used was suitable, EIS data were obtained for the TQ-NiFe photoanode at the designated applied potential and under illumination. As shown in Supplementary Fig. 19, the fitting curves (solid lines) provided good agreement with the experimental EIS data (hollow symbols). Subsequently, we fitted the current density with ${J}=k{[{a}_{{{{{{\rm{OH}}}}}}^{-}}]}^{\alpha}$ (i.e., $\log {J}={{{{{\rm{log}}}}}}{k}+{\alpha }\log [{a}_{{{{{{\rm{OH}}}}}}^{-}}]$) at different illumination intensities to explore the effect of the hole concentration on the reaction rate constant (Supplementary Fig. 20), where $[{{a}}_{{{{{{{\rm{OH}}}}}}}^{-}}]$ and *α* are the hydroxide ion activity and the reaction order for the hydroxide ion activity, respectively. The increasing trend for the calculated reaction rate constant *k* with increasing illumination intensity is shown in Fig. 2d. Many holes were photogenerated in the TQ layer at stronger illumination intensities. The photogenerated holes were adequately and efficiently transferred along the energy level gradient to participate in the interface reaction, thereby leading to a continuous increase in the reaction rate constant. Similarly, to explore the effect of the hydroxide concentration on the reaction rate constant, we fitted the current density with ${J}={k}{[{{{{{\rm{hole}}}}}}]}^{{\beta }}$ (i.e., $\log {J}\,=\,\log ({k})+{\beta }\,\log [{{{{{\rm{hole}}}}}}]$) at different hydroxide ion concentrations (Supplementary Fig. 21), where $[{{{{{\rm{hole}}}}}}]$ is the surface trapped hole concentration calculated from the electrochemical impedance spectroscopy (EIS) measurements (Supplementary Fig. 22) and β is the reaction order of the surface trapped holes. The variations of the fitted reaction rate constant as a function of the hydroxide concentration are shown in Fig. 2e. The reaction rate constant *k* gradually increased with increasing hydroxide concentrations. The larger reaction rate constant was mainly due to the strong catalytic ability of the NiFe LDHs, which adsorbed many hydroxide ions that participated in reactions with the photogenerated holes, thereby accelerating the reaction kinetics^33,34^. In addition, the hydroxide ion activity and surface trapped hole concentrations verified the kinetics of the as-fabricated catalysts in a practical alkaline solution (Supplementary Figs. 23–26). Furthermore, we depicted the photocurrent density of the TQ-NiFe as a function of the two factors, as shown in Fig. 2f, by fitting the data into the kinetic model defined by Eq. 1:

1a $${J}=k{[{a}_{{{{{{\rm{OH}}}}}}^{-}}]}^{\alpha }{[{{{{{\rm{hole}}}}}}]}^{\beta }$$

1b $$\log {J}=\,\log k+\alpha \,\log [{a}_{{{{{{\rm{OH}}}}}}^{-}}]+\beta \,\log [{{{{{\rm{hole}}}}}}]$$

where *J* is the current density at 1.23 *V*_RHE_, and *k* is the water oxidation rate constant. As a result, the TQ-NiFe exhibited a larger calculated reaction rate constant of 0.457; this confirmed the faster reaction kinetics, which originated from efficient transfer of more photogenerated holes excited into the TQ layer and the higher reactivity of the cocatalyst NiFe LDHs.

### Photoenhanced electrocatalytic oxygen evolution reaction (OER)

**Fig. 3:**
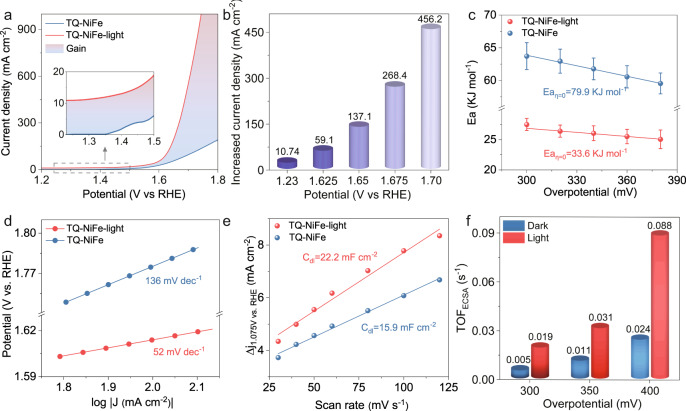
Activity investigations performed during the electrocatalytic OER process under irradiation. **a** OER electrocatalytic properties of the TQ-NiFe with and without illumination. **b** Increased current densities for TQ-NiFe under irradiation. **c** Activation energies for the OER with TQ-NiFe. **d** Tafel plots for TQ-NiFe with and without illumination. **e** Plots of the charging current density versus scan rate for TQ-NiFe. **f** ECSA-normalized TOFs calculated from the current.

After the PEC performance tests, we investigated the OER performance of the as-prepared catalysts in a 1 M KOH solution (pH = 13.6) and with high potentials. As shown in Fig. 3a, b, under illumination, the TQ-NiFe reached a current density of 10 mA cm^−2^ below the theoretical potential (1.23 V vs. RHE) due to the contribution of the photocurrent. In comparison to traditional PEC catalysts, the TQ-NiFe exhibited a remarkable gain in the current density at high anodic potentials. During illumination, the current density of the TQ-NiFe exhibited a 2.81-fold enhancement from 42.1 to 118.2 mA cm^−2^ at 1.65 V and a 4.46-fold increase from 103.9 to 463.8 mA cm^−2^ at 1.75 V. Moreover, gas chromatographic measurements confirmed the high faradaic efficiency of approximately 99.7% for O_2_ formation at 1.43 V (an overpotential of 200 mV) (Supplementary Fig. 27). With the increased potential, this large increase in current density under illumination already far exceeded the traditional photocurrent. Traditional PECs exhibit saturation photocurrents. Further increases in the anodic potential lead to sharp rises in the dark-state current density, and this dominates the total current so a significant enhancement of the current is not observed. Thus, it can be concluded that the improvement seen under illumination probably arose from the positive impact of light on the activity of the catalyst.

Then, the activation energy (*E*_a_), electrochemical kinetics and activity per catalytic site of the TQ-NiFe were studied under irradiation or dark conditions. Figure 3c shows the apparent electrochemical *E*_*a*_ of the as-prepared TQ-NiFe, which is a key parameter used to assess the barriers to the OER^38,39^. As shown in Fig. 3c, the *E*_*a*_ extracted for TQ-NiFe under illumination was ~32.5 kJ mol^−1^, which was much lower than those for the dark reaction (79.9 kJ mol^−1^) and the reported transition metal hydroxide/oxide OER catalysts^38,40,41^ and comparable to those of noble metal catalysts (30.8 kJ mol^−1^)^42^. The lower *E*_*a*_ under illumination suggested that the interaction of the TQ and NiFe-LDH could have greatly reduced the reaction barrier under illumination. In addition, Tafel plots were generated to assess the kinetic behaviour (Fig. 3d). The TQ-NiFe catalyst showed a significantly reduced Tafel slope of 78 mV dec^−1^ under illumination compared to 177 mV dec^−1^ without illumination, implying that there was a change in the rate-determining step. In contrast, there were no obvious changes in either the *E*_*a*_ or Tafel values for the NiFe-LDH after illumination, which ruled out the influence of light on the NiFe-LDH itself (Supplementary Figs. 28–32). Furthermore, the TQ-NiFe catalyst exhibited long catalytic durability after a 100 h test and 500 cycles under illumination. The morphology and chemical composition were also well maintained after the stability test (Supplementary Figs. 35–37).

To minimize the effects of the electrochemically active surface area (ECSA), the normalized turnover frequencies (TOF_ECSA_) and mass activities of the samples were determined from their ECSAs and loading amounts (Supplementary Figs. 38–41)^43–45^. As shown in Fig. 3e, the slope of the TQ-NiFe composite catalyst exhibited a C_dl_ of 22.2 mF cm^−2^ under illumination, higher than the *C*_dl_ of 15.9 mF cm^−2^ seen under dark conditions. Figure 3f presents the TOF_ECSA_ values of the TQ-NiFe for different applied overpotentials under illumination or in the dark. Notably, the normalized TOF_ECSA_ values for the TQ-NiFe at overpotentials of 300 mV, 350 mV and 400 mV and under illumination were 3.6, 2.8, and 3.7 times higher than those seen under dark conditions. However, the ECSA and TOF_ECSA_ values of the bare NiFe-LHD were unchanged after illumination (Supplementary Figs. 40 and 41). These results demonstrated that illumination greatly improved the intrinsic activity of the TQ-NiFe owing to its unique structure and composition. Moreover, the enhancement induced by illumination was confirmed by using the FTO substrate (Supplementary Fig. 42).

### In situ XAS analysis

**Fig. 4:**
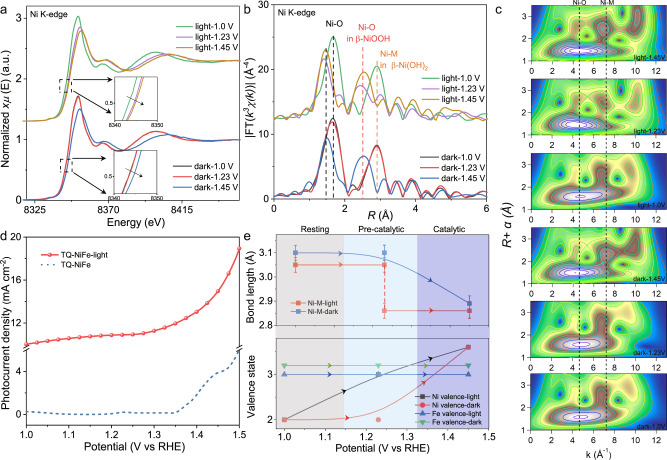
XAFS spectroscopy. **a** *Operando* Ni K-edge XANES spectra for TQ-NiFe at different potentials without and with illumination. **b** FT-EXAFS and WT-EXAFS data for TQ-NiFe under different potentials without and with illumination, which were not corrected for phase shifts. **c** Corresponding wavelet transforms for the k^3^-weighted Ni K-edge EXAFS signals of TQ-NiFe. **d** LSV curves for TQ-NiFe from 1.0–1.5 V without and with illumination. **e** Changes in the bond lengths and valence states of Ni and Fe as a function of the applied potential for TQ-NiFe without and with illumination. The valence states were obtained by comparing the Ni/Fe K-edge energy positions with those of standard references for known oxidation states.

To reveal the underlying mechanism of the photoenhanced OER activity, *operando* XAS was performed to detect the changes occurring in the chemical states of both Ni and Fe in the TQ-NiFe during the OER under illuminated and dark conditions (Fig. 4). First, the in situ Fe K-edge X-ray absorption near-edge structure (XANES) of the TQ-NiFe spectra indicated that the absorption edge showed no observed shift during the entire OER process (Supplementary Fig. 43), suggesting there was no valence change for the Fe^3+^ cations. In contrast, the absorption edge in the Ni K-edge spectra displayed an obvious positive shift as the applied bias was increased from 1.0 to 1.45 V (Fig. 4a), which indicated an increase in the oxidation state of the Ni species, in agreement with our previous results for NiFe-MOFs^14^. In particular, with an applied potential at 1.23 V under illumination, the Ni K-edge XANES peaks shifted to higher energies (red solid curve in Fig. 4a), while the peaks did not shift until a potential of 1.45 V under the dark conditions (yellow solid curve in Fig. 4a); this implied that the increase in the Ni oxidation state occurred at a lower potential under illumination, verifying that an interaction between the TQ and NiFe-LDH increased the oxidation states of the active sites for the OER.

Additionally, the FT-EXAFS results showed negative shifts of the Ni-O and Ni-M (M=Ni/Fe) peaks with increasing applied potential (Fig. 4b), indicating shrinkage of the bonds in the TQ-NiFe. Specifically, without illumination, the Ni-O and Ni-M peaks were located at 1.75 and 2.85 Å at 1.23 V, respectively. When the potential was increased to 1.45 V, the Ni–O and Ni–M peaks shifted to 1.55 and 2.53 Å, which corresponded to a structural transition from NiFe(OH)_2_ to NiFeOOH^14^. There were no obvious changes at higher potentials. However, when light was introduced together with electrocatalytic potentials, the Ni–O and Ni–M peaks shifted as the potential was increased to 1.23 V (Fig. 4b), which indicated that NiFe(OH)_2_ was oxidized to NiFeOOH at a low catalytic potential. These structural changes were also confirmed by an EXAFS wavelet transform (WT) analysis, which revealed the appearance of a stronger WT intensity maximum at 1.23 V under illumination; in contrast, the same phenomenon at occurred 1.45 V without illumination (Fig. 4c). An additional quantitative analysis showed that the changes in the Ni valence states and Ni–M bond lengths were completely synchronous with the increase in the photocurrent density (Fig. 4d, e, Supplementary Figs. 44 and 45, Supplementary Tables 3 and 4), confirming that NiFeOOH was the real active species. In attempting to explore the difference in the Ni^3+/4+^ species caused by light, we also obtained the Ni K-edge XANES spectrum of pure NiFe (Supplementary Fig. 46). The NiFe sample did not exhibit a similar shift of the absorption edge under illumination, which resembled the behaviour of the TQ-NiFe without illumination. Therefore, the in situ XAS results for TQ-NiFe and NiFe revealed that the photogenerated holes facilitated a structural transition from NiFe(OH)_2_ to NiFeOOH and oxidation of the Ni^2+^ ions to active Ni^4+^, which increased the OER catalytic activity.

## Discussion

In summary, we developed a three-dimensional TQ-NiFe photoelectroanode with semiconductor-electrocatalyst nano interfaces for use in catalyzing the water oxidation reaction. The TQ-NiFe composite photoelectrode showed high-performance PEC properties and dramatically photoenhanced EC OER properties at high anodic potentials. The underlying mechanism for the improvement was confirmed by *operando* XAS, which indicated that light greatly accelerated the OER kinetics by forming more Ni^3+/4+^ active species at lower anodic potentials, thereby leading to a lower Tafel slope and activation energy. The present findings provide a novel route for the development of water splitting catalytic systems.

## Methods

Preparation of TiO_2_-CdS/CdSe-MoS_2_ on carbon cloth: Titanium dioxide (TiO_2_) nanorods were grown on carbon cloth with a hydrothermal method^20^. In a typical growth process, 15 mL of deionized water was mixed with 15 mL of concentrated hydrochloric acid (36–38% by weight) in a 50 mL Teflon-lined stainless steel autoclave. Then, 0.75 mL of titanium tetrachloride (TiCl_4_) was added to the mixture, and the mixture was stirred well. After that, a piece of glass with carbon cloth fixed on one side was placed at an angle against the wall of the Teflon-liner with the carbon cloth side facing down. Then, the autoclave was heated at 150 °C for 12 h in an electric oven and cooled to room temperature. After the hydrothermal treatment, the carbon cloth was removed, washed with deionized water and dried in air. Then, the prepared carbon cloth containing TiO_2_ nanorods was annealed at 450 °C for 30 min (denoted as TiO_2_).

CdS/CdSe quantum dots were grown on the TiO_2_ nanorods by successive ionic layer adsorption and reaction (SILAR)^23^. The methanol solution of 0.1 M Cd(NO_3_)_2_ was employed as a Cd^2+^ source. The solution of DI water and methanol (volume ratio of 3:7) of 0.1 M Na_2_S·9H_2_O was used as the S^2−^ source. The Se^2−^ precursor was prepared by dissolving 0.15 M Na_2_SO_3_ and 0.1 M selenium powder in DI water and then heating at 80 °C for 12 h under a nitrogen atmosphere. The TiO_2_ samples were alternately immersed in the following solutions: Cd^2+^ solution for 1 min, methanol (removal of excess Cd^2+^), S^2−^ or Se^2−^ solution for 1 min and methanol (removal of excess S^2−^ or Se^2−^). After carried out 6 cycles of the above process, the CdS/CdSe quantum dots-sensitized TiO_2_ nanorods on the carbon cloth (denoted as TiO_2_–CdS/CdSe) were obtained. Subsequently, the TiO_2_–CdS/CdSe sample was dipped into an ammonium thiomolybdate solution (1 mg/1 ml) and then annealed at 425 °C for 30 min under a reduction atmosphere (7% H_2_ in Ar), and thus a protective layer of MoS_2_ was formed (denoted TQ).

### Preparation of TiO_2_-CdS/CdSe-MoS_2_-NiFe on carbon cloth

The NiFe-LDH on TQ was synthesized via a simple hydrothermal method. In a typical procedure, Ni(NO_3_)_2_·6H_2_O (0.625 mmol), Fe(NO_3_)_3_·9H_2_O (0.625 mmol) and urea (7.5 mmol) were added into a mixed solution of ethylene glycol/water (7:1 v/v, 30 mL) under vigorous stirring until they dissolved completely. Then, the autoclave with the TQ sample and the above precursor was maintained at 160 °C for 12 h and naturally cooled to room temperature. The resulting composite was rinsed with distilled water and anhydrous ethanol and dried at room temperature (denoted as TQ-NiFe). The NiFe-LDH on carbon cloth and TiO_2_ (denoted as NiFe and T-NiFe, respectively) were prepared with the same synthetic method.

### Characterization

X-ray diffraction (XRD) patterns of samples were collected at room temperature with a PANalytical X-pert diffractometer (PANalytical, Netherlands) using Cu Kα radiation at 40 kV and 40 mA over the 2*θ* range 5–80°. Scanning electron microscopy (SEM) images were obtained with a Hitachi S-4800 microscope. Transmission electron microscopy (FEI Tecnai G2 F20, 200 kV) was used to observe the morphologies of the samples. The transmission electron microscopy (TEM) samples were prepared by immersing a 300 mesh copper grid with an ultrathin carbon film into a solution containing various samples. The HAADF-STEM images and EDS maps were generated at 300 kV by using an aberration-corrected FEI Titan Themis G2 with a spatial resolution up to 60 pm. The convergence semiangle for imaging was 30 mrad, and the collection semiangle snap was 39 to 200 mrad for HAADF. The chemical states of Ni, Fe, Cd, S, Se, Mo and Ti in the composite were evaluated with X-ray photoelectron spectroscopy (XPS) (PerkinElmer Physics PHI 5300).

The PEC and electrochemical measurements were performed with a three-electrode configuration. A 1 M KOH solution served as the electrolyte for the OER. A mercuric oxide electrode was used as the reference electrode, and platinum foil was used as the counter electrode. All other samples were fabricated with well-defined areas of 1 cm^2^. The measurements were carried out by applying an external bias to the cell with an electrochemical station (CHI660E workstation) operated at a scan rate of 2 mV s^−1^. Electrochemical impedance spectra (EIS) were collected with the same instrument. A 150 W Xe solar simulator (Newport 94021 A) with an AM 1.5 G filter was used as the light source. Incident-photon-to-current-conversion efficiency (IPCE) spectra were measured with a QE/IPCE Measurement Kit (Crowntech QTest Station 1000AD) with a tungsten halogen lamp (CT-TH-150), a calibrated silicon diode and a monochromator (Crowntech QEM24-S 1/4 m). In the electrocatalytic process, the IR drop was compensated at 95% with the positive feedback model.

### XAS data collection and analyses

The Ni and Fe *K*-edge XAS data were acquired under *operando* conditions at beamline 1W1B for the Beijing Synchrotron Radiation Facility (BSRF); a Si (111) double-crystal monochromator (operated at 2.5 GeV with a maximum current of 250 mA) was used. A homemade electrochemical cell was employed for the *operando* XAS experiments under the sensitive fluorescence model, and this was combined with a computer-controlled electrochemical analyser and a tungsten halogen lamp. Details for the configuration of the cell and the test method are provided in Supplementary Fig. 43. An as-prepared thin carbon cloth was used as the working electrode, a Pt wire was used as the counter electrode and an Ag/AgCl electrode was used as the reference electrode. During the measurements, various potentials were applied to the working electrode in the 1 M KOH electrolyte.

The XAS raw data were then background-subtracted, normalized and Fourier transformed with the standard procedures in the ATHENA programme^46^. Least-squares curve fitting analyses were applied to the EXAFS *χ*(*k*) data by using the ARTEMIS programme based on the standard EXAFS equation. The theoretical scattering amplitudes, phase shifts and the photoelectron mean free paths were calculated with the ab initio code FEFF 8.5^47^. All fits were performed in the *R* space with *k*-weight of 3. The best-fit results are shown in Supplementary Figs. 44 and 45, and the fitting parameter values are listed in Supplementary Tables 3 and 4.

## Supplementary information

Supplementary Information

## Data Availability

The data that support the findings of this study are available in the supplementary material of this article.

## Code availability

The code that supports the findings of this work is available from the corresponding authors upon reasonable request.

## References

[1] Liu J (2018). Ultrathin amorphous cobalt-vanadium hydr(oxy)oxide catalysts for the oxygen evolution reaction. Energy Environ. Sci..

[2] Wang C (2014). Enhancing visible-light photoelectrochemical water splitting through transition-metal doped TiO_2_ nanorod arrays. J. Mater. Chem. A.

[3] Guo B (2018). Facile integration between Si and catalyst for high-performance photoanodes by a multifunctional bridging layer. Nano Lett..

[4] Hao N (2018). In situ hybridization of an MXene/TiO_2_/NiFeCo-layered double hydroxide composite for electrochemical and photoelectrochemical oxygen evolution. RSC Adv..

[5] Shakeel M, Arif M, Yasin G, Li B, Khan HD (2019). Layered by layered Ni-Mn-LDH/g-C_3_N_4_ nanohybrid for multi-purpose photo/electrocatalysis: morphology controlled strategy for effective charge carriers separation. Appl. Catal. B Environ..

[6] Lv J (2018). A photo-responsive bifunctional electrocatalyst for oxygen reduction and evolution reactions. Nano Energy.

[7] Wang G (2011). Hydrogen-treated TiO_2_ nanowire arrays for photoelectrochemical water splitting. Nano Lett..

[8] Kim JS, Kim B, Kim H, Kang K (2018). Recent progress on multimetal oxide catalysts for the oxygen evolution reaction. Adv. Energy Mater..

[9] Jamesh M-I, Sun X (2018). Recent progress on earth abundant electrocatalysts for oxygen evolution reaction (OER) in alkaline medium to achieve efficient water splitting - a review. J. Power Sources.

[10] Tahir M (2017). Electrocatalytic oxygen evolution reaction for energy conversion and storage: a comprehensive review. Nano Energy.

[11] Yang X (2014). Enabling practical electrocatalyst-assisted photoelectron-chemical water splitting with earth abundant materials. Nano Res..

[12] Roger I, Shipman MA, Symes MD (2017). Earth-abundant catalysts for electrochemical and photoelectrochemical water splitting. Nat. Rev. Chem..

[13] Zhao S (2016). Ultrathin metal-organic framework nanosheets for electrocatalytic oxygen evolution. Nat. Energy.

[14] Zhao S (2020). Structural transformation of highly active metal-organic framework electrocatalysts during the oxygen evolution reaction. Nat. Energy.

[15] Jiang C, Moniz SJA, Wang A, Zhang T, Tang J (2017). Photoelectrochemical devices for solar water splitting - materials and challenges. Chem. Soc. Rev..

[16] Niu F (2020). Hybrid photoelectrochemical water splitting systems: from interface design to system assembly. Adv. Energy Mater..

[17] Zhang Z, Wang P (2012). Optimization of photoelectrochemical water splitting performance on hierarchical TiO_2_ nanotube arrays. Energ. Environ. Sci..

[18] Seger B (2013). Using TiO_2_ as a conductive protective layer for photocathodic H_2_ evolution. J. Am. Chem. Soc..

[19] Seh ZW (2012). Janus Au-TiO_2_ photocatalysts with strong localization of plasmonic near-fields for efficient visible-light hydrogen generation. Adv. Mater..

[20] Aydil BLAES (2019). Growth of oriented single-crystalline rutile TiO_2_ nanorods on transparent conducting substrates for dye-sensitized solar cells. J. Am. Chem. Soc..

[21] Schneider J (2014). Understanding TiO_2_ photocatalysis: mechanisms and materials. Chem. Rev..

[22] Sheng P (2013). A novel method for the preparation of a photocorrosion stable core/shell CdTe/CdS quantum dot TiO_2_ nanotube array photoelectrode demonstrating an AM 1.5 G photoconversion efficiency of 6.12%. J. Mater. Chem. A.

[23] Liu XY, Chen Z, Li WX, Cao MS (2017). Distinctly improved photocurrent and stability in TiO_2_ nanotube arrays by ladder band structure. J. Phys. Chem. C..

[24] Xu X, Hu J, Yin Z, Xu C (2014). Photoanode current of large-area MoS_2_ ultrathin nanosheets with vertically mesh-shaped structure on indium tin oxide. ACS Appl. Mater. Inter..

[25] Liu KK (2012). Growth of large-area and highly crystalline MoS_2_ thin layers on insulating substrates. Nano Lett..

[26] Ho TA (2019). Heterojunction photoanode of atomic-layer-deposited MoS_2_ on single-crystalline CdS nanorod arrays. ACS Appl. Mater. Interfaces.

[27] Zhou W (2019). 5.1% efficiency of Si photoanodes for photoelectrochemical water splitting catalyzed by porous NiFe (oxy)hydroxide converted from NiFe oxysulfide. Chem. Commun..

[28] Bhat SSM (2019). Substantially enhanced photoelectrochemical performance of TiO_2_ nanorods/CdS nanocrystals heterojunction photoanode decorated with MoS_2_ nanosheets. Appl. Catal. B Environ..

[29] Han HS (2018). Boosting the solar water oxidation performance of a BiVO_4_ photoanode by crystallographic orientation control. Energ. Environ. Sci..

[30] Qi XP (2014). High-performance n-Si/alpha-Fe_2_O_3_ core/shell nanowire array photoanode towards photoelectrochemical water splitting. Nanoscale.

[31] Zhou SQ (2019). High-performance photoelectrochemical water splitting of BiVO_4_@Co-MIm prepared by a facile in-situ deposition method. Chem. Eng. J..

[32] Wang SC (2018). New iron-cobalt oxide catalysts promoting BiVO_4_ films for photoelectrochemical water splitting. Adv. Funct. Mater..

[33] Doyle RL, Lyons ME (2013). An electrochemical impedance study of the oxygen evolution reaction at hydrous iron oxide in base. Phys. Chem. Chem. Phys..

[34] Zhang YC (2018). Rate-limiting O-O bond formation pathways for water oxidation on hematite photoanode. J. Am. Chem. Soc..

[35] Le Formal F (2015). Rate law analysis of water oxidation on a hematite surface. J. Am. Chem. Soc..

[36] Louie MW, Bell AT (2013). An investigation of thin-film Ni-Fe oxide catalysts for the electrochemical evolution of oxygen. J. Am. Chem. Soc..

[37] Yeo BS, Bell AT (2012). In situ raman study of nickel oxide and gold-supported nickel oxide catalysts for the electrochemical evolution of oxygen. J. Phys. Chem. C..

[38] Zhang B (2016). Homogeneously dispersed multimetal oxygen-evolving catalysts. Science.

[39] Zheng X (2018). Theory-driven design of high-valence metal sites for water oxidation confirmed using in situ soft X-ray absorption. Nat. Chem..

[40] Swierk JR, Klaus S, Trotochaud L, Bell AT, Tilley TD (2015). Electrochemical study of the energetics of the oxygen evolution reaction at nickel iron (oxy)hydroxide catalysts. J. Phys. Chem. C..

[41] Hu WC (2019). Plasmonic hot charge carriers activated Ni centres of metal–organic frameworks for the oxygen evolution reaction. J. Mater. Chem. A.

[42] Yao YC (2019). Engineering the electronic structure of single atom Ru sites via compressive strain boosts acidic water oxidation electrocatalysis. Nat. Catal..

[43] Ni Y (2017). Construction of hierarchically porous graphitized carbon-supported NiFe layered double hydroxides with a core-shell structure as an enhanced electrocatalyst for the oxygen evolution reaction. Nanoscale.

[44] Yu L (2017). Cu nanowires shelled with NiFe layered double hydroxide nanosheets as bifunctional electrocatalysts for overall water splitting. Energ. Environ. Sci..

[45] McCrory CC (2015). Benchmarking hydrogen evolving reaction and oxygen evolving reaction electrocatalysts for solar water splitting devices. J. Am. Chem. Soc..

[46] Ravel B, Newville M (2005). ATHENA, ARTEMIS, HEPHAESTUS: data analysis for X-ray absorption spectroscopy using IFEFFIT. J. Synchrotron Radiat..

[47] Rehr JJ, Albers RC (2000). Theoretical approaches to x-ray absorption fine structure. Rev. Mod. Phys..

