# Supplementary material for: A semiconductor-electrocatalyst nano interface constructed for successive photoelectrochemical water oxidation
Source: Nat Commun. 2023 May 4;14:2574. doi: 10.1038/s41467-023-38285-z (PMC10160110; doi:10.1038/s41467-023-38285-z)
Supplement: Supplementary file 1 — Supplementary Information [file 41467_2023_38285_MOESM1_ESM.pdf]

SUPPLEMENTAL INFORMATION

A Semiconductor-Electrocatalyst Nano Interface Constructed for Successive Photoelectrochemical Water Oxidation

Zilong Wu[1#], Xiangyu Liu[1#], Haijing Li[2#], Zhiyi Sun[1], Maosheng Cao[1], Zezhou Li[3], Chaohe Fang[4], Jihan Zhou[3], Chuanbao Cao[1], Juncai Dong[2*], Shenlong Zhao[5*] and Zhuo Chen[1*]

[1]Energy & Catalysis Center, Department of Materials Physics and Chemistry, School of Materials Science and Engineering, Beijing Institute of Technology, Beijing 100081, China.

[2]Beijing Synchrotron Radiation Facility, Institute of High Energy Physics, Chinese Academy of Sciences, Beijing, China.

[3]College of Chemistry and Molecular Engineering, Beijing National Laboratory for Molecular Sciences, Peking University, Beijing, P. R. China.

[4]CNPC Research Institute of Petroleum Exploration & Development, Beijing, 100083, P. R. China.

[5]School of Chemical and Biomolecular Engineering, The University of Sydney, Sydney, New South Wales, Australia.

[#]These authors contributed equally to this work.

[*]Corresponding authors: zchen@bit.edu.cn

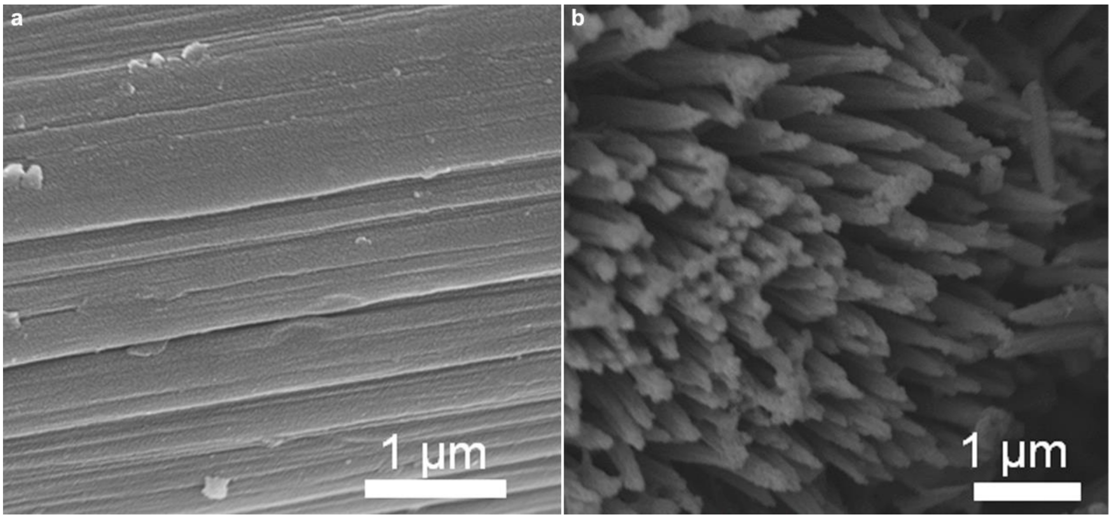

Supplementary Figure 1. SEM images. SEM images of pure carbon cloth (**a**) and TiO_2 (**b**).

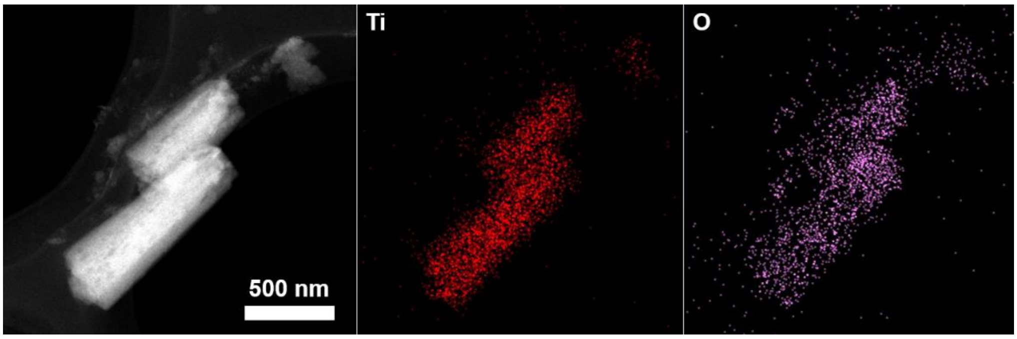

Supplementary Figure 2. HRTEM-EDS measurement. HRTEM-EDS elemental mapping images of Ti and O in the TiO_2.

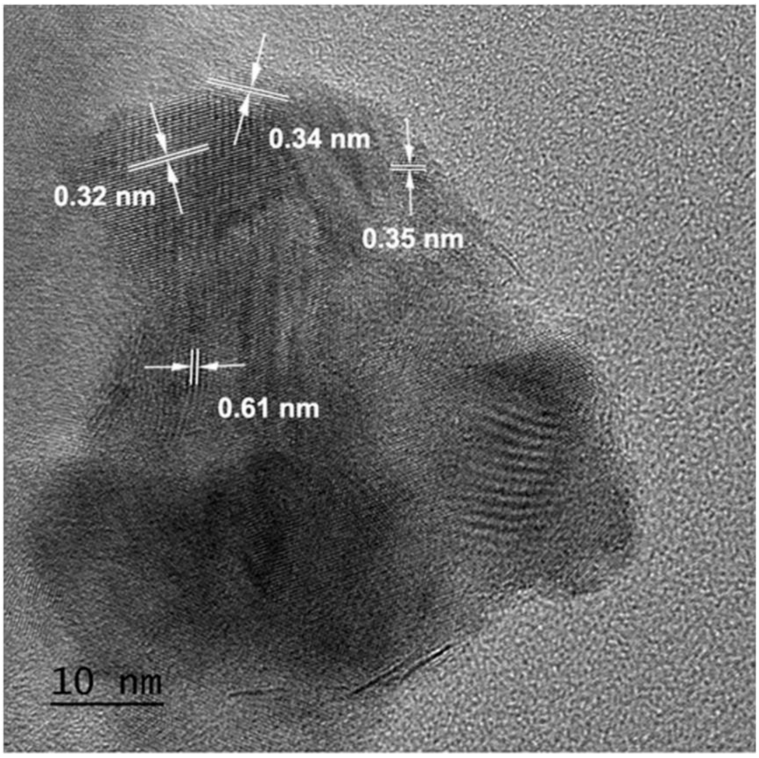

Supplementary Figure 3. HRTEM images. TEM image of TQ

As shown in Supplementary Figure 3, the lattice fringe spacing of 0.32 nm corresponds to the interplanar distance of the (110) plane in rutile TiO_2. The lattice fringe spacing of 0.34 and 0.35 nm corresponds to (111) plane in cubic structure of CdS and (111) plane in cubic structure of CdSe respectively, revealing that cadmium chalcogenide nanoparticles with a diameter of ~4 nm indeed are attached to TiO_2 nanorods surface. Also, the lattice fringe spacing of 0.61 nm is observed corresponding to the (002) plane of hexagonal MoS_2.

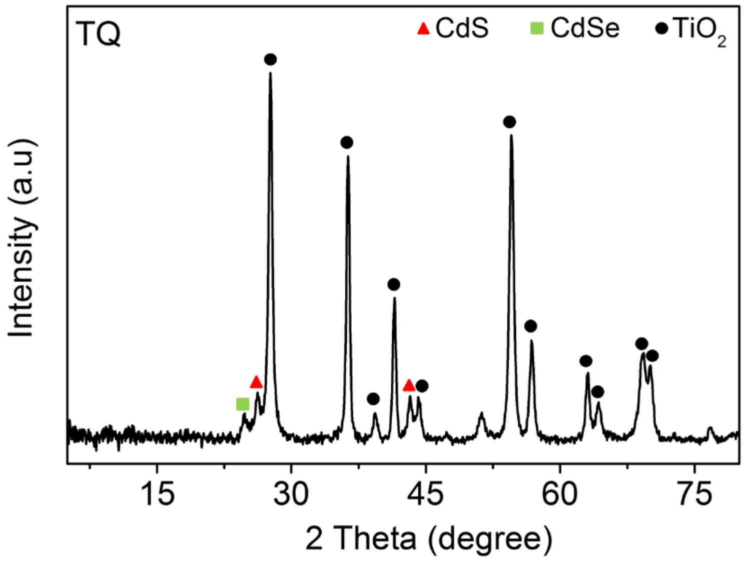

Supplementary Figure 4. XRD measurement. XRD spectrum of TQ.

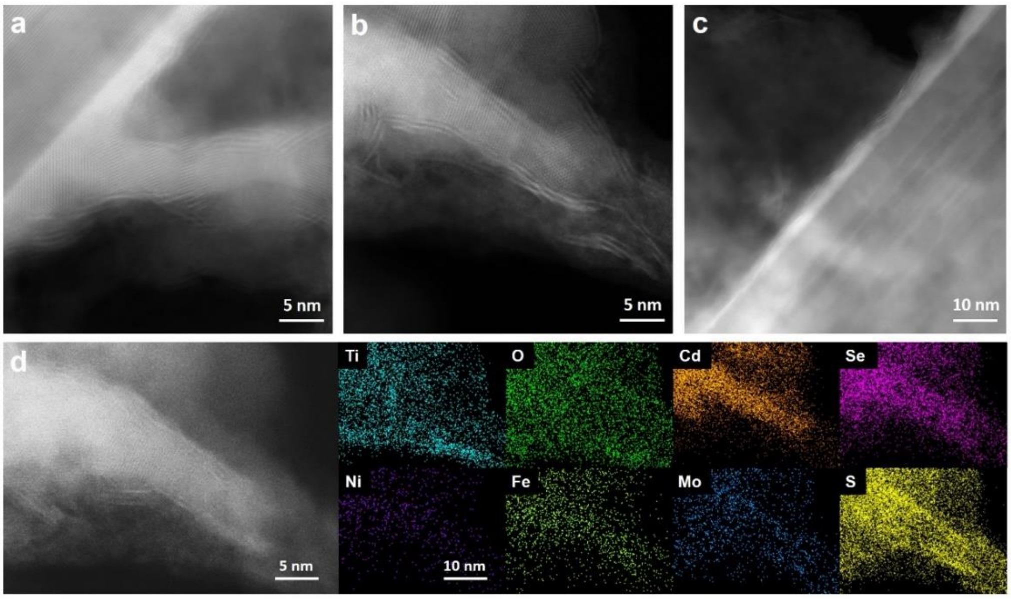

Supplementary Figure 5. **HAADF-STEM and EDS images** (a-c). Atomic resolution HAADF-STEM images of the TQ-NiFe. (d). EDS mapping of the TQ-NiFe structure.

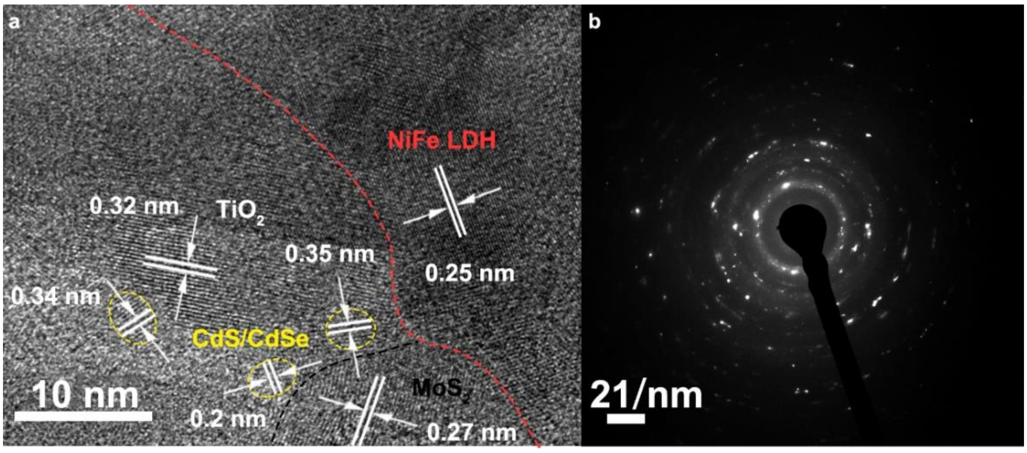

Supplementary Figure 6. HRTEM measurement. HRTEM image of the as-prepared TQ-NiFe (**a**). The selected area electron diffraction (SAED) of TQ-NiFe (**b**).

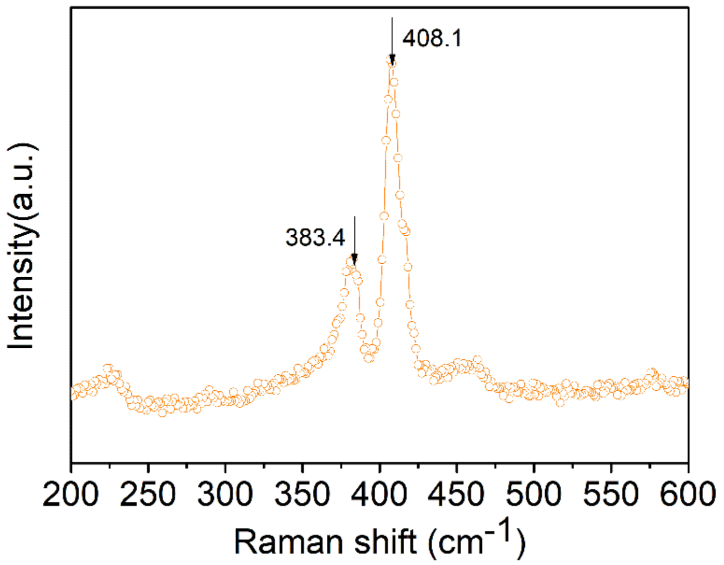

Supplementary Figure 7. Raman spectroscopy. Raman spectrum for the as-prepared TQ.

As the diffraction peak of MoS_2 is too weak to be observed, the Raman spectroscopy of the TQ is displayed in Supplementary Figure 7 to verify the existence of MoS_2, which shows the two typical Raman peaks of MoS_2 at 383.4 cm^{-1} and 408.1 cm^{-1}.

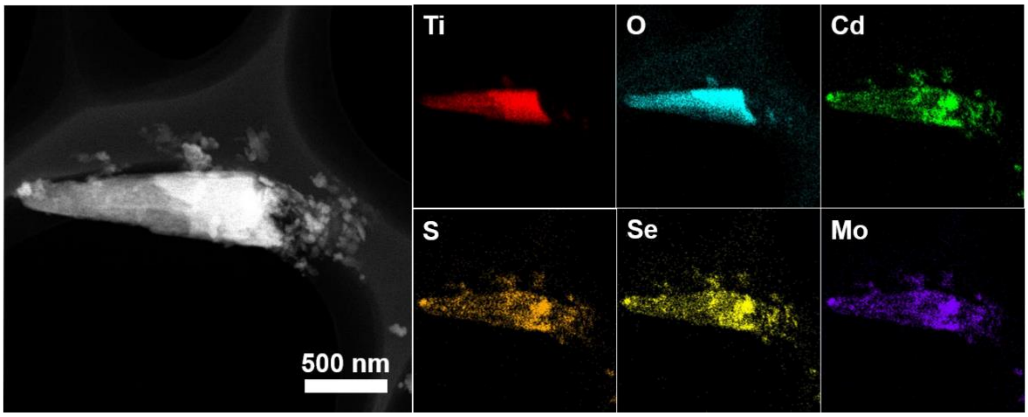

Supplementary Figure 8. HRTEM-EDS measurement. HRTEM-EDS elemental mapping images of Ti, O, Cd, S, Se and Mo in the TQ.

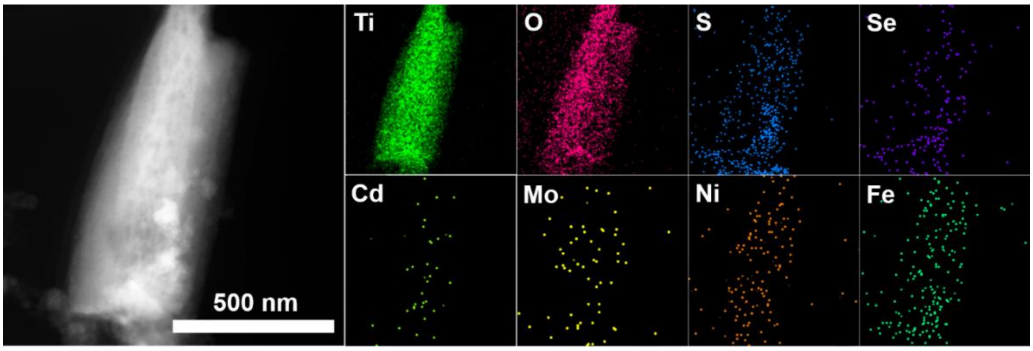

Supplementary Figure 9. HRTEM-EDS measurement. HRTEM-EDS elemental mapping images of Ti, O, Cd, S, Se, Mo, Ni and Fe in the as-prepared TQ-NiFe.

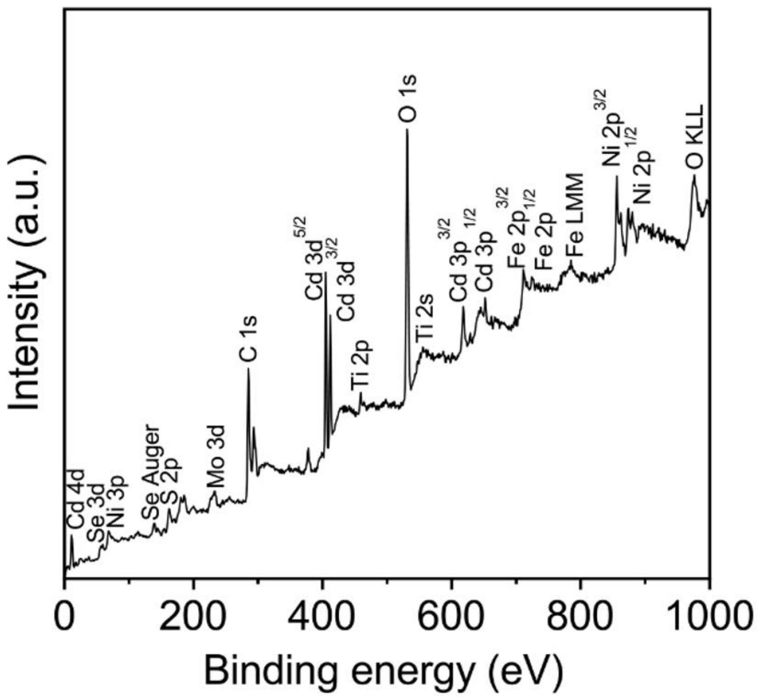

Supplementary Figure 10. XPS measurement. XPS spectrum of TQ-NiFe.

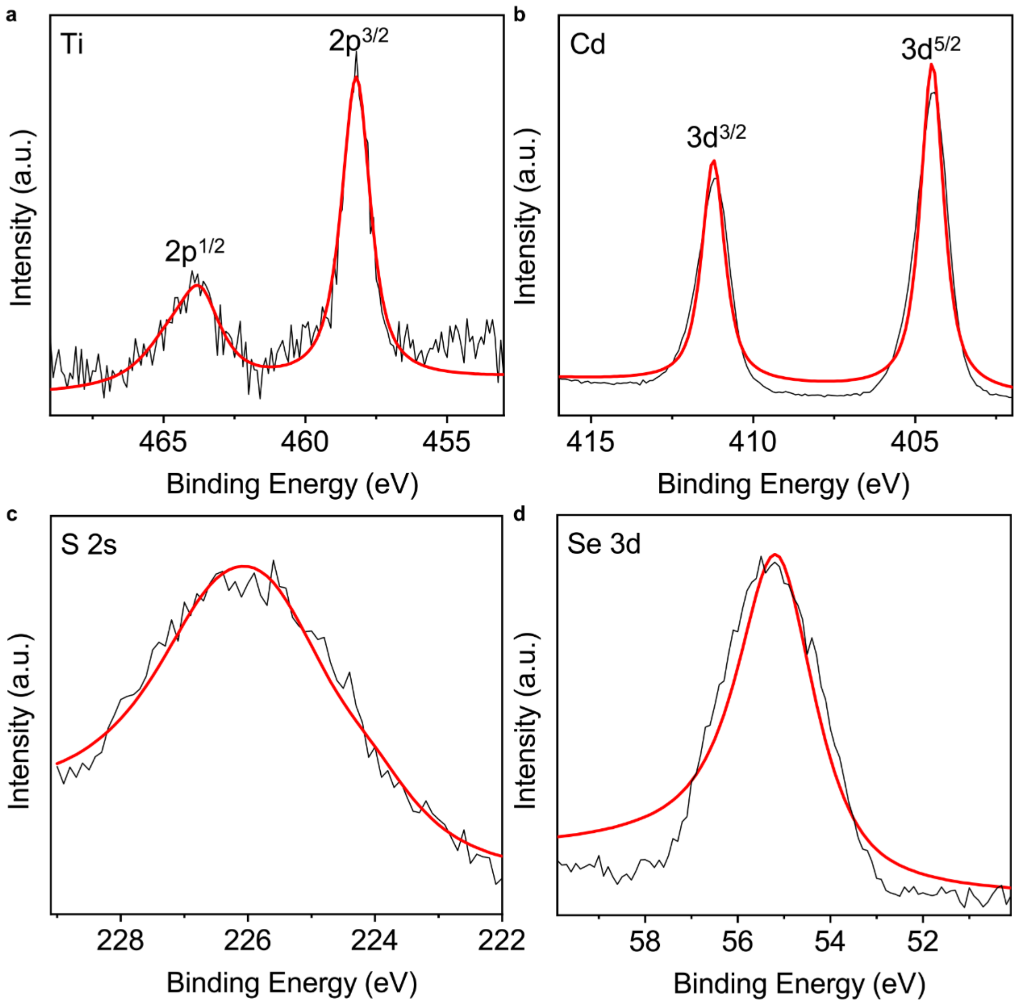

Supplementary Figure 11. XPS measurement. XPS spectra of Ti 2p (**a**), Cd 3d (**b**), S 2s (**c**) and Se 3d (**d**) in TiO_2/CdS-CdSe.

In XPS spectrum, Ti $2p^{1/2}$ and Ti $2p^{3/2}$ peaks in TiO_2 were obtained at 464.4 eV and 458.8 eV. In XPS spectrum of TiO_2/CdS-CdSe, peaks of Ti 2p (463.8 eV and 458.2 eV) undergo a little negative shift, and the appearance of Cd 3d (411.2 eV and 404.4 eV), S 2s (225.9 eV) and Se 3d (55.3 eV) indicates the existence of Cd, S and Se related to CdS/CdSe quantum dots in the composite.

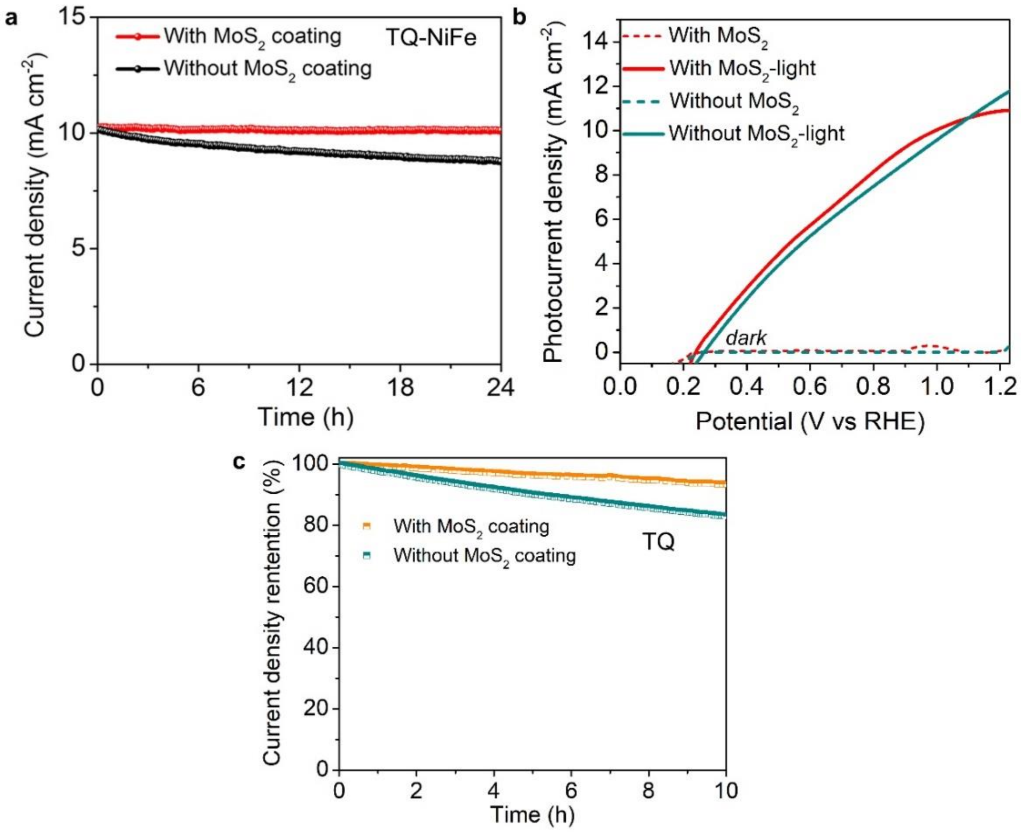

Supplementary Figure 12. Stability test. (**a**) Photocurrent density stability of TQ-NiFe with or without MoS_2 layer. (**b**) Photoelectrochemical properties of TQ-NiFe with or without MoS_2 layer. (**c**) Photocurrent density stability of TQ with or without MoS_2 layer.

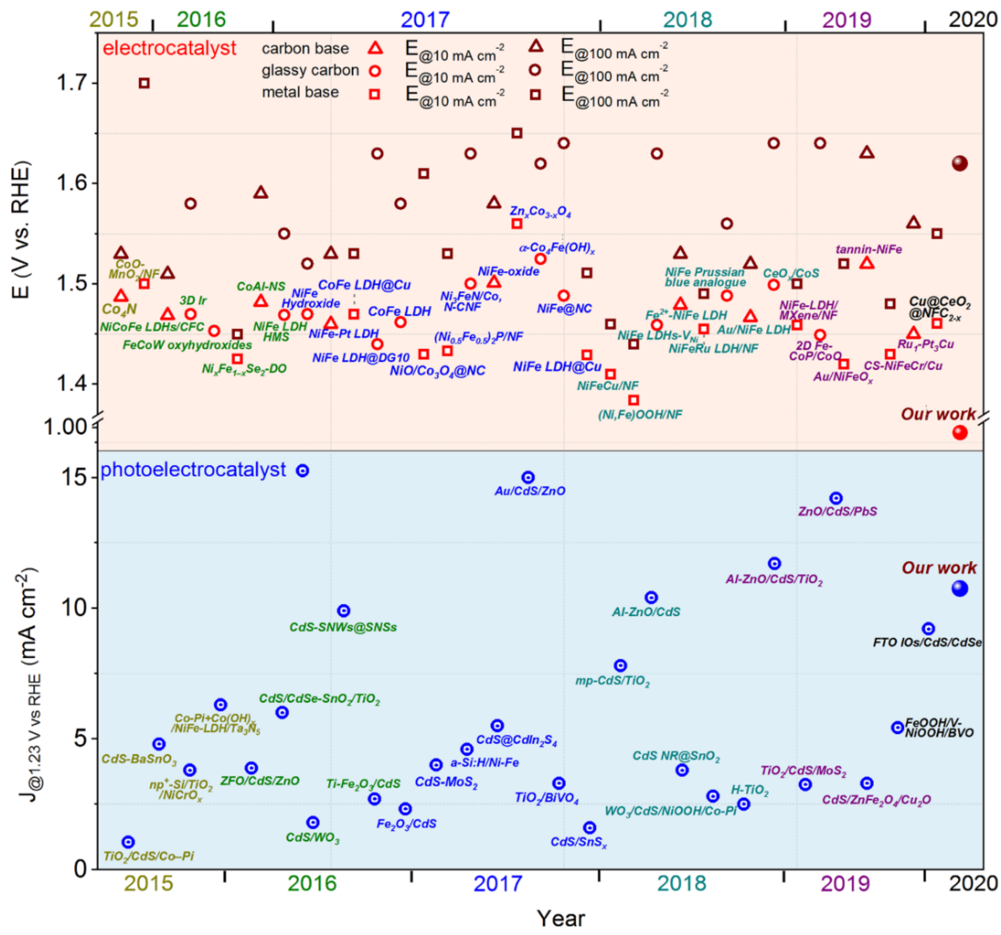

Supplementary Figure 13. Comparison of the water splitting performance. Comparison of the water-splitting performance (potential for 10 mA cm^{-2} and 100 mA cm^{-2}) for the electrocatalysts (pink area). Comparison of the water-splitting performance (photocurrent density at 1.23 V vs RHE) for the photoelectrocatalysts (blue area).

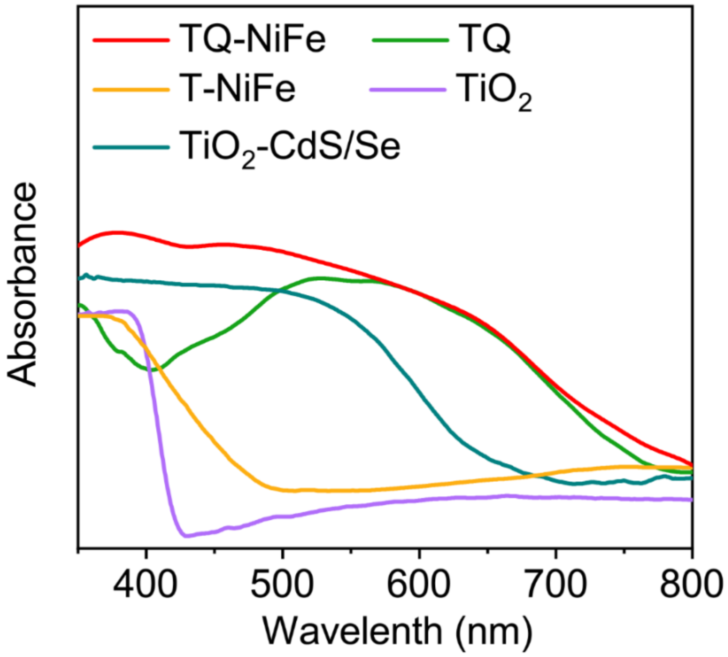

Supplementary Figure 14. UV-vis absorption spectrum testing. UV-vis absorption spectra of TQ-NiFe, TQ, T-NiFe, TiO_2 and TiO_2-CdS/CdSe.

As typical II-VI semiconductors, the bandgaps of CdS and CdSe are 2.4 eV and 1.7 eV, respectively, corresponding to absorption edges of 517 nm and 729 nm. Moreover, the bandgap and absorption properties can be easily tuned by their composition and size. Considering the materials in our photoanode, we believe that the response in the range of 400-500 nm comes from the CdS/CdSe quantum dot layer, which can be further confirmed by its absorption spectra as shown in supplementary Figure 14. Compared with TiO_2 or T-NiFe samples, the CdS/CdSe quantum dots can significantly broaden the visible light absorption range of 400-600 nm. Based on the absorption results, we also observed the improvement of the IPCE in the range of 400-500 nm.

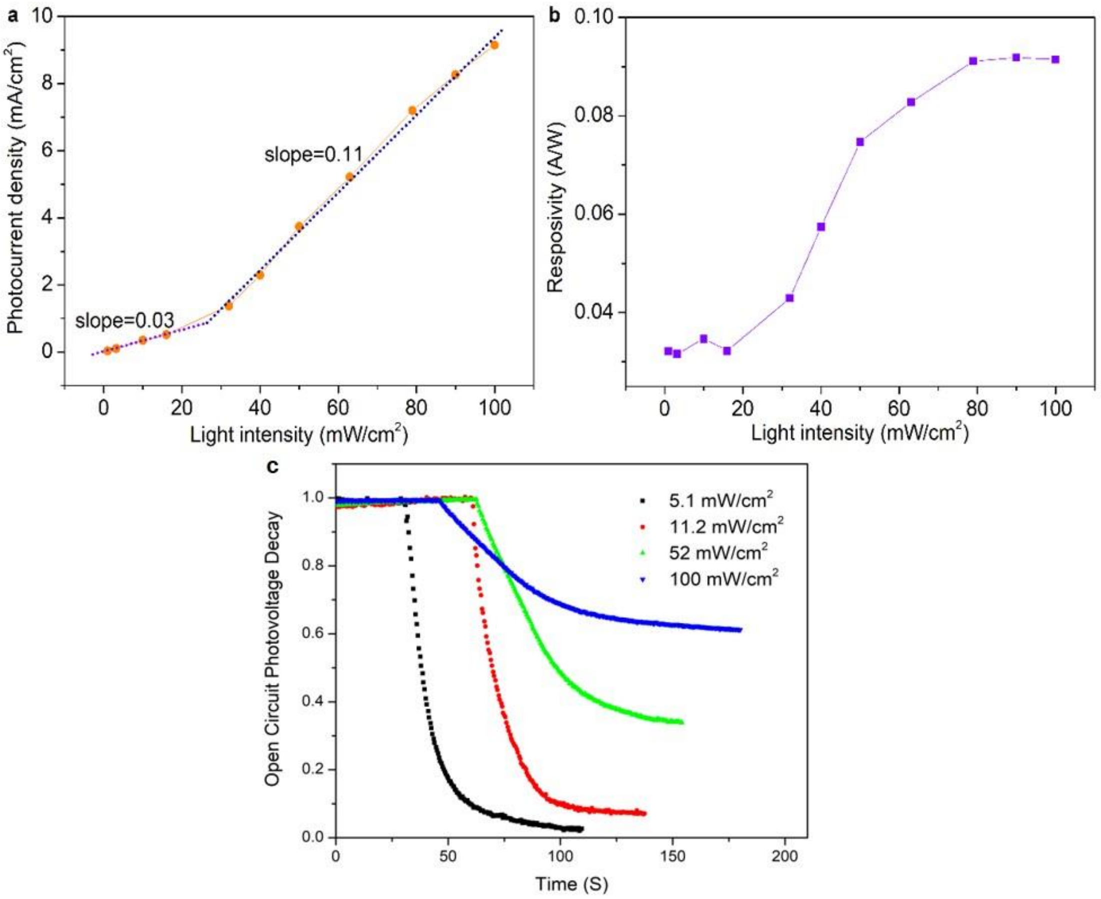

Supplementary Figure 15. Light intensity effect. (**a**) Variation of the photocurrents with the incident optical power. (**b**) Responsivity vs optical power density for the TQ-NiFe photoanode at 0.9 V vs. RHE. (**c**) The decay of photovoltage with time at different light intensity.

The effect of light intensity on the photocurrents of TQ-NiFe was investigated at the collected external bias (0.9 V vs. RHE). A 150 W Xe solar simulator (Newport 94021A) with neutral density filters for a range of 1~100 mW/cm^2 was used as light source. The light intensity was double-checked by a standard Si solar cell (SRC-1000-TC-QZ, SN 10510-0309) and a power meter (LPE-1A). Supplementary Figure 15a shows two clear domains with different slopes (0.03 and 0.12) can be generated as the photocurrent varies. The photocurrent increases relatively slow at low power densities (below 30 mW/cm^2) due to the contribution of trap recombination. On contrary, trap filling can enhance the lifetime of the photogenerated charge carriers and can improve the quantum yield at higher light intensities.[1-3] With increased intensity, the traps can

be saturated, leading to the enhanced photocurrent. Furthermore, the relationship for responsivity vs. optical power was plotted in Supplementary Figure 15b. Here, the responsivity is expressed by the standard equation ($R=I_{Photocurrent}/P$), where $I_{Photocurrent}$ and P are the photocurrent and light intensity respectively. Obviously, the responsivity starts to be small and accompanied by a little fluctuation, and gradually increases and reaches to the maximum at 80 mW/cm^2. Based on the above results, the discrepancy between the photocurrents and the integration of the IPCE results is ascribed to the trap filling effect under illumination with low optical power. In fact, this phenomenon generally exits in various semiconductor materials (TiO_2 [4-6], GaN [7] etc.) and devices, such as solar cells [4,5], photodetectors [7] and photoandoes. [8]

For a PEC cell, the photocurrent is equal to the carrier generation rate multiplied by the collection probability, in which the carrier generation rate can be expressed as $G = \alpha I_0 e^{-\alpha x}$. The trapped photocarrier cannot contribute to the photocurrent. The trap state will significantly decrease the carrier lifetime and thus the collection probability. Therefore, the trap state in a photoanode is strongly related to the carrier lifetime, which can be characterized by measuring their open-circuit photovoltage decay (OCPD). In order to identify the effect of the trap state on the photocurrent, we carried out the OCPD measurements, which consist of turning off illumination at a steady-state and monitoring the subsequent decay of photovoltage, V_{oc}, with time (Supplementary Figure 15). The V_{oc} decay rate is directly related to the carrier lifetime by the following equation:

$$\tau = \frac{k_B T}{e}\left(\frac{dV_{oc}}{dt}\right)^{-1}$$

where τ is the potential dependent photocarrier lifetime, k_B is Boltzmann's constant, T is the temperature, e is the charge of a single electron, and V_{oc} is the open-circuit voltage at time t. Clearly, as shown in Supplementary Figure 15 the photocarrier lifetime increases with increasing light intensity. The results showed relatively short lifetime at lower light intensity. With the increase of light intensity, the trap state is gradually saturated, leading to longer lifetime.

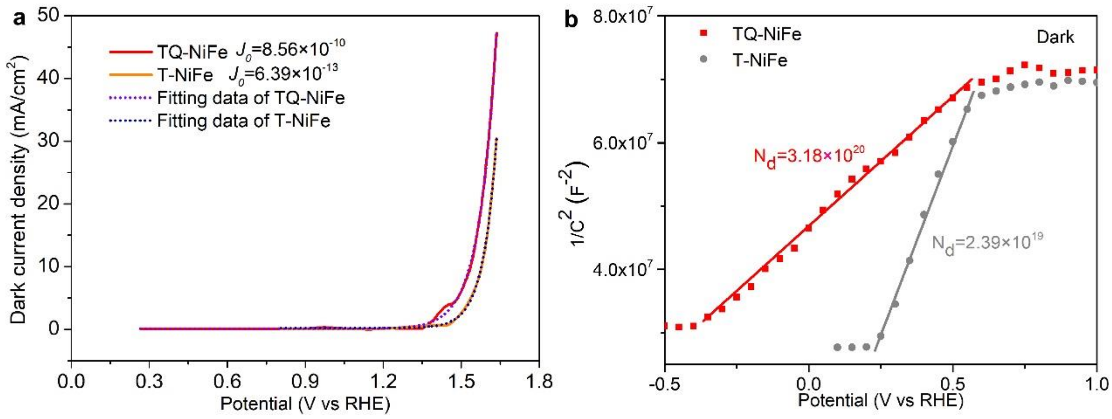

Supplementary Figure 16. The dark current and Mott-Schottky plots. (**a**) Dark current curves of TQ-NiFe and T-NiFe and the corresponding fitting curves based on Butler-Volmer equation. (**b**) Mott-Schottky plots of TQ-NiFe and T-NiFe. Dots are experimental data and lines are fitting data.

As shown in Supplementary Figure 16a, the dark current of the TQ-NiFe is 0.09 mA /cm^2, significantly lower than the photocurrent.

To clarify why the T-NiFe has a smaller potential window compared to the TQ-NiFe, we use Butler-Volmer kinetics to model electron transfer between the catalyst and the redox couple through the standard equation ($J = J_0(e^{qV/2kT} - e^{-qV/2kT})$). In the high anode potential region, the cathode current in Butler-Volmer equation can be ignored, leading to a simplified expression: $J = J_0 e^{qV/2kT}$. For the semiconductor/electrocatalyst system, the expression can be given as $J = J_0 e^{q(V-Voc)/2kT}$, where V_{oc} is the open-circuit voltage. Based on this, Thomas J. Mills et al. developed a theory of charge transfer at semiconductor-catalyst interfaces to elucidate the current-potential behavior of semiconductor-catalyst-solution system [9]. They found that the onset potential will increase (i.e. I-V curve shifts to the right) as J_0 is decreased. Therefore, the equation $J = J_0 e^{qV/2kT}$ was adopted to fit the dark current curves of TQ-NiFe and T-NiFe. As shown in Supplementary Figure 16a, two fitting data (J_0) are 8.56 x 10^{-10} for TQ-NiFe and 6.39 x 10^{-13} for T-NiFe, respectively. The exchange current density of the TQ-NiFe is three orders of magnitude higher than that of the TQ-NiFe, thereby leading to a lower onset potential. Additionally, from the Mott-Schottky plots of TQ-NiFe and T-NiFe in Supplementary Figure 16b, one can obtain

that the carrier concentration of the TQ-NiFe is 13 times higher than that of the TQ-NiFe. The increased carrier concentration after introducing CdS/CdSe quantum dots will cause Femi level rise, thereby leading to that the TQ-NiFe has a larger V_{oc} compared to the T-NiFe. Therefore, the larger exchange current density and open-circuit voltage of the TQ-NiFe should be responsible for its larger potential window.

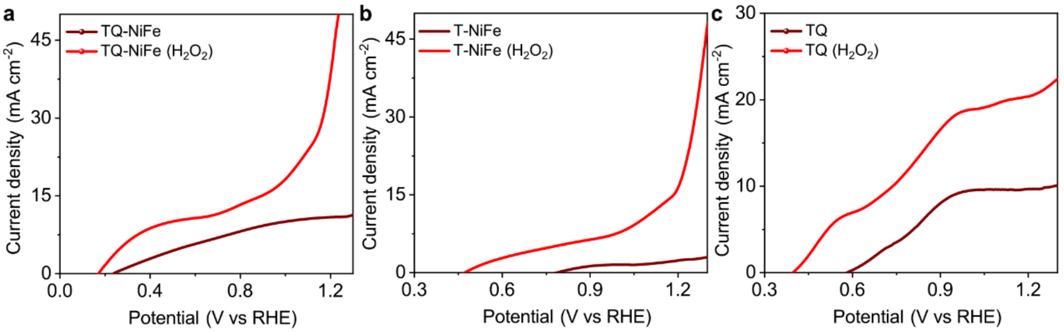

Supplementary Figure 17. Polarization curve testing. Polarization curves of the TQ-NiFe in the presence of 0.5 M H_2O_2 solution (**a**). Polarization curves of the T-NiFe in the presence of 0.5M H_2O_2 solution (**b**). Polarization curves of the TQ in the presence of 0.5M H_2O_2 solution (**c**).

To evaluate the surface charge transfer behavior in photoelectrocatalysis of the TQ-NiFe, TQ and T-NiFe, we also measured the photocurrent density in the presence of 0.5M H_2O_2 solution as hole scavenger, as shown in Supplementary Figure 17. The composite electrodes show higher photocurrent density in the presence of 0.5M H_2O_2 solution, resulting from that the charge transfer efficiency can be assumed as 100%. The photocurrent can be described as: $J = J_{abs}\times\eta_{sep}\times\eta_{trans}$, the charge transfer efficiency (η_{trans}) values can be calculated as J_{water}/J_{H2O2}.

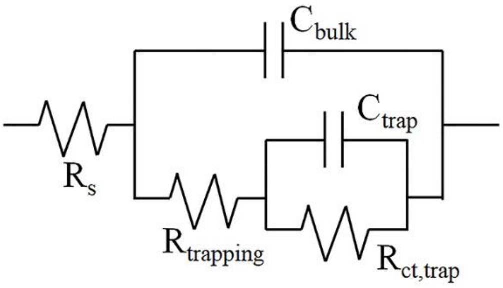

Supplementary Figure 18. Equivalent circuit model. R_s is defined as the series resistance, C_{bulk} represents the capacitance of charge accumulation in the space charge layer, $R_{trapping}$ represents the resistance of holes trapping at the surface states, $R_{ct, trap}$ represents the resistance of charge transfer from the surface states to the solution, and C_{trap} represents the capacitance associated with charge accumulation on the surface states.

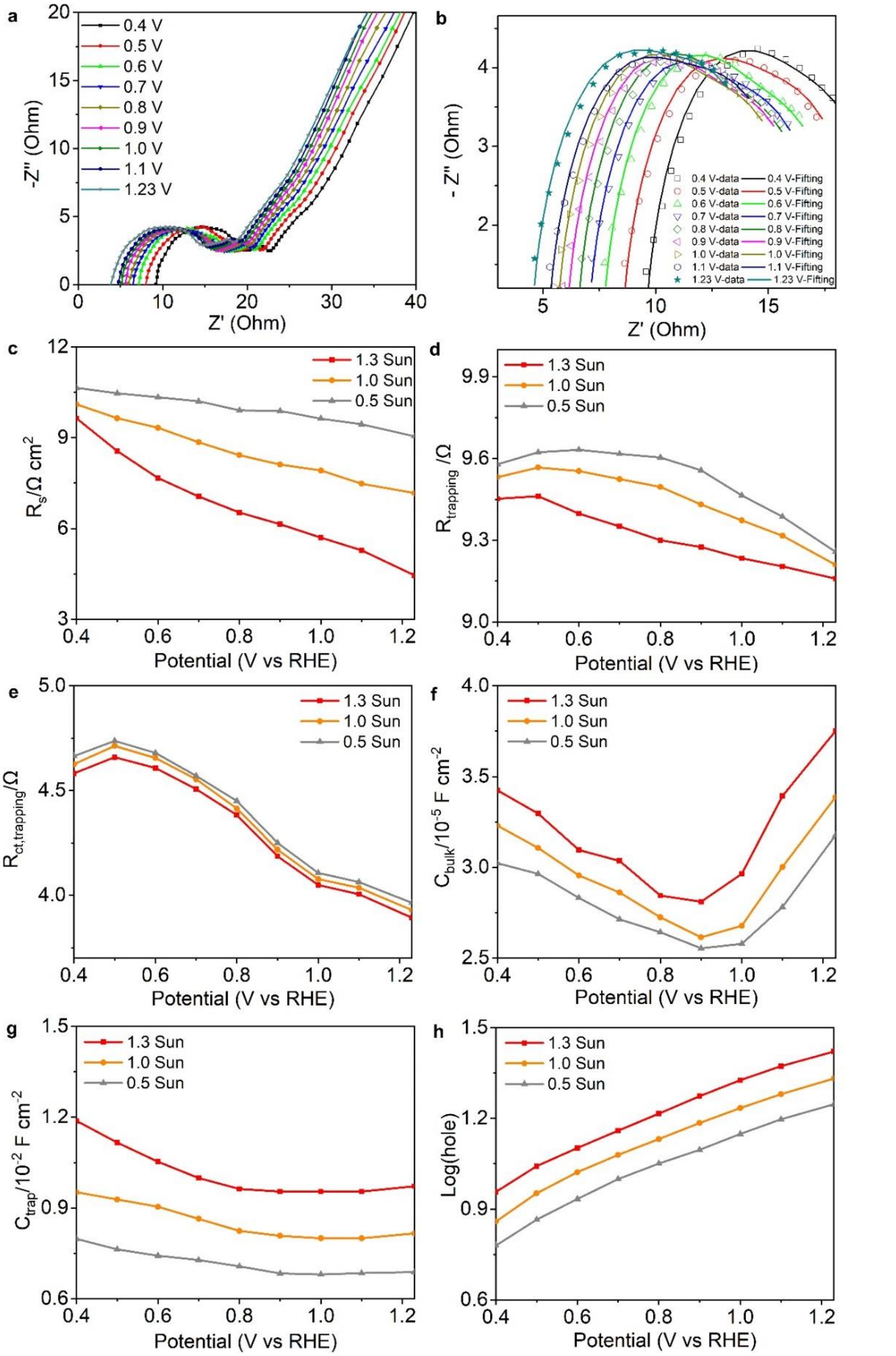

Supplementary Figure 19. EIS data of the TQ-NiFe photoanode at the designated applied potential under illumination (**a**) Nyquist plots of the TQ-NiFe photoanode at the designated applied potential under 130 mW/cm^2. (**b**) Fitting EIS data of the TQ-NiFe photoanode via equivalent circuit model. (**c-e**) Resistances (R_s, $R_{trapping}$ and $R_{ct,trap}$)

and (**f-g**) capacitances (C_{bulk} and C_{trap}) parameters associated with the charge trapping and transfer at different applied potentials and light intensities that are determined from the EIS spectra. (**h**) The calculated hole densities at different applied potentials and light intensities.

To verify whether the equivalent circuit model (Supplementary Fig. 18) we use is suitable, we measured EIS data of the TQ-NiFe photoanode at the designated applied potential under illumination (Supplementary Fig. 19a). Impedance data were gathered using a 5 mV amplitude perturbation of between 100000 and 0.1 Hz. The applied potential range is from 0.4 V to 1.23V (V vs RHE). And, the intensity of light source was set to 50 mW cm^{-2}, 100 mW cm-2 and 130 mW cm-2, respectively. Impedance fitting result between 10 0000 and 1000 Hz is shown in Supplementary Fig. 19b. As we can see, the fitting curves (solid lines) are in good agreement with the experimental EIS data (hollow symbols), suggesting that the equivalent circuit model we use is suitable. Furthermore, the charge trapping ($R_{trapping}$, C_{bulk}) and transfer (Rct, trap, C_{trap}) values derived from the EIS results are plotted according to the corresponding equivalent circuit (Supplementary Fig. 19b-19g). The decreased $R_{trapping}$ value in TQ-NiFe illustrates the restrained recombination of trapping electrons and holes (Supplementary Fig. 19d), while the enhanced C_{bulk} value indicates the increased carrier density after light intensity increase (Supplementary Fig. 19f). Besides, the strong correlation among the decreased $R_{ct,\ trap}$ and increased C_{trap} illustrates the accumulation of holes in surface states at the photoanode/electrolyte interface (Supplementary Fig. 19e and 19g). Despite the C_{bulk} and C_{trap} show similar tendencies along with applied potentials, C_{trap} is three orders of magnitude higher than C_{bulk}. The larger value in C_{trap} suggests that most of the photoinduced charges mainly locate on the surface states. The density of surface trapped holes can be calculated by the following equation: $[hole]=C_{trap}\times V_{appl}\times R_{ct,trap}/(R_s+R_{trapping}+R_{ct,trap})/S$. The calculated hole densities at different applied potentials and light intensities are shown in Supplementary Fig. 19h.

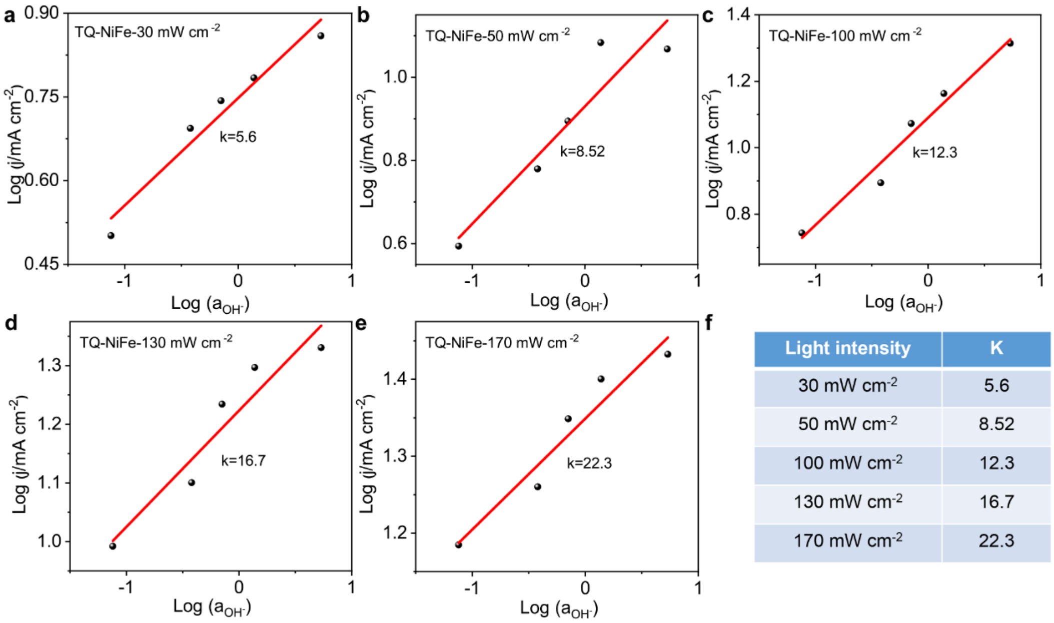

Light intensity	K
30 mW cm^{-2}	5.6
50 mW cm^{-2}	8.52
100 mW cm^{-2}	12.3
130 mW cm^{-2}	16.7
170 mW cm^{-2}	22.3

Supplementary Figure 20. Dynamic analysis. Fitted dynamic line of TQ-NiFe with hydroxide ion activity under 30 mW cm^{-2} (**a**), 50 mW cm^{-2} (**b**), 100 mW cm^{-2} (**c**), 130 mW cm^{-2} (**d**) and 170 mW cm^{-2} (**e**). The fitted reaction rate constant k under different illumination intensity (**f**).

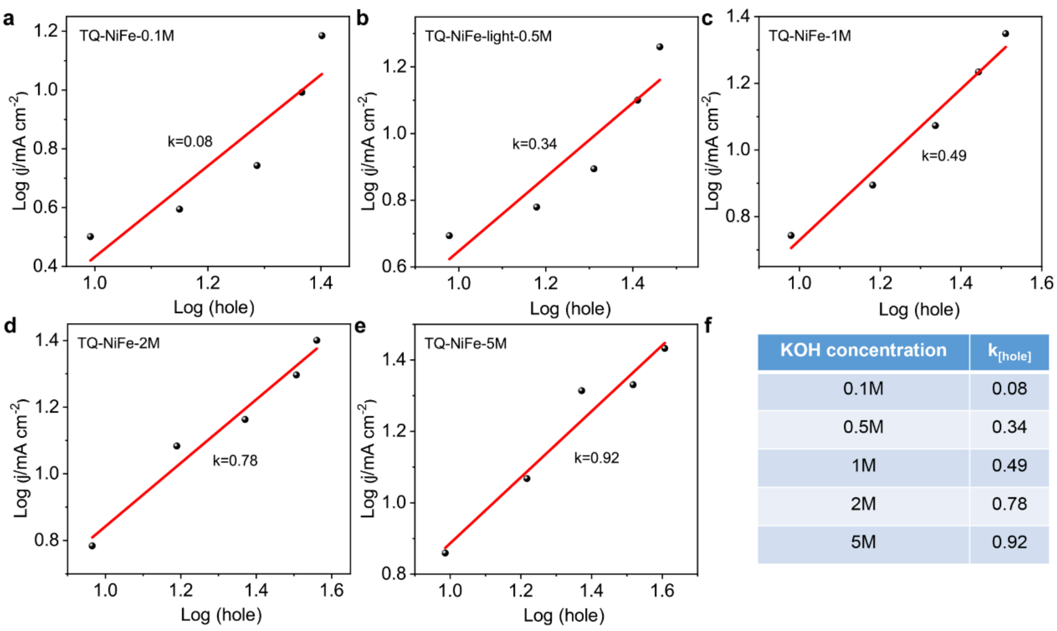

f

KOH concentration	$k_{[hole]}$
0.1M	0.08
0.5M	0.34
1M	0.49
2M	0.78
5M	0.92

Supplementary Figure 21. Dynamic analysis. Fitted dynamic line of TQ-NiFe with hole concentration in 0.1 M (**a**), 0.5 M (**b**), 1 M (**c**), 2 M (**d**) and 5 M (**e**). The fitted reaction rate constant $k_{[hole]}$ in different KOH concentrations (**f**).

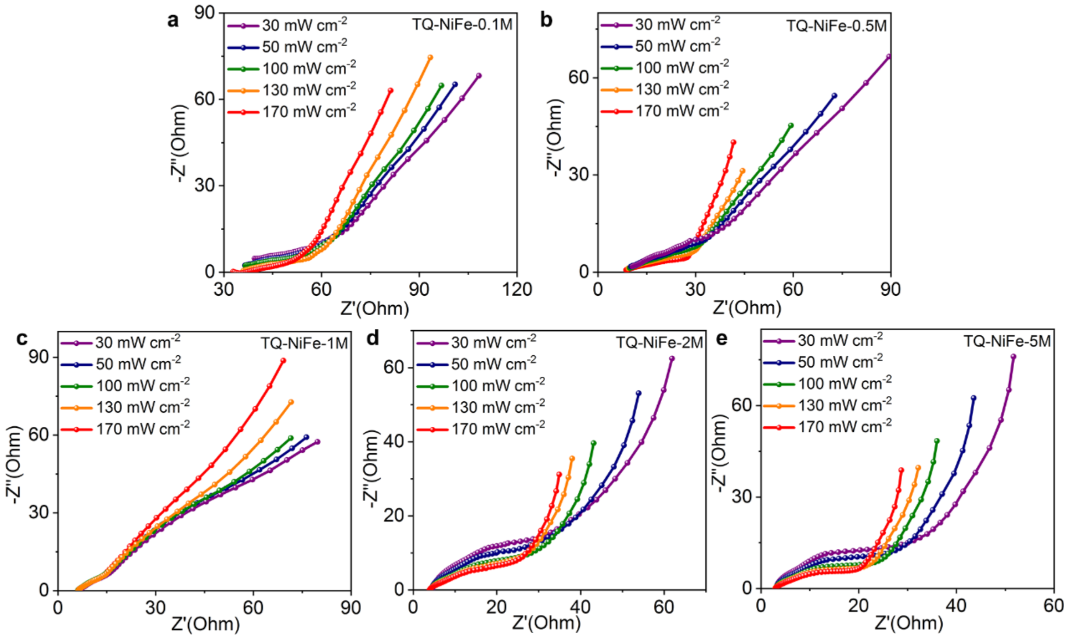

Supplementary Figure 22. EIS analysis. EIS data of TQ-NiFe obtained under illumination with the applied potential of 1.23 V vs RHE in 0.1 M (**a**), 0.5 M (**b**), 1 M (**c**), 2 M (**d**) and 5 M (**e**).

The Nyquist plots exhibit two semicircles for this model. The high-frequency semicircle represents the process of hole trapping by surface states (hole accumulation at the surface), while the radius of low-frequency semicircle reflects the process of the interfacial holes transfer to OH^-. The density of surface trapped holes can be calculated by the following equation:

$$[\text{hole}] = C_{trap} \times V_{appl} \times \frac{R_{ct,trap}}{R_s + R_{trapping} + R_{ct,trap}} / S$$

where V_{appl} is the applied potential, $R_{trapping}$ is the resistance in surface hole trapping, C_{trap} is the charges accumulated at surface states, $R_{ct,trap}$ is the resistance of interfacial charge transfer and S is the active area of the electrode.

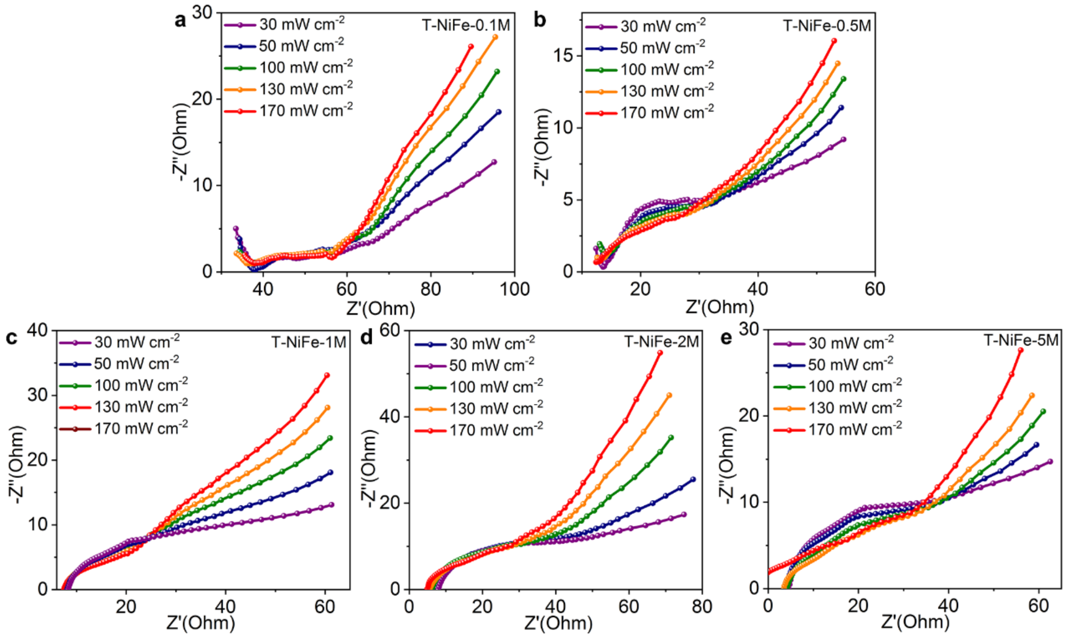

Supplementary Figure 23. EIS analysis. EIS data of T-NiFe obtained under illumination with the applied potential of 1.23 V vs RHE in 0.1 M (**a**), 0.5 M (**b**), 1 M (**c**), 2 M (**d**) and 5 M (**e**).

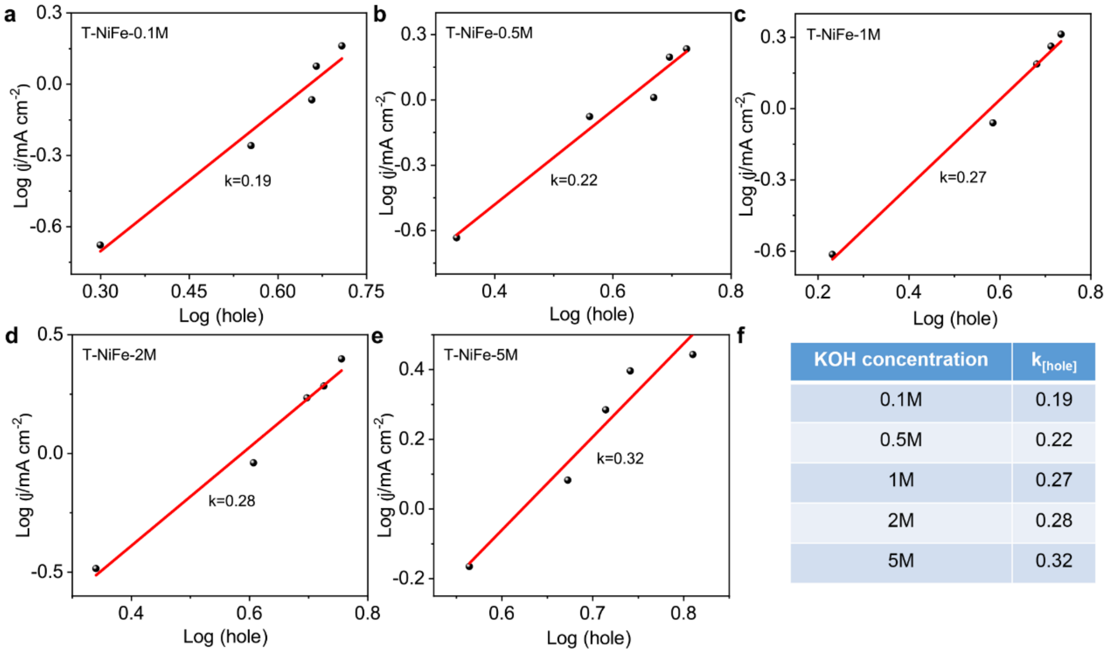

f

KOH concentration	$k_{[hole]}$
0.1M	0.19
0.5M	0.22
1M	0.27
2M	0.28
5M	0.32

Supplementary Figure 24. Dynamic analysis. Fitted dynamic line of T-NiFe with hole concentration in 0.1 M (**a**), 0.5 M (**b**), 1 M (**c**), 2 M (**d**) and 5 M (**e**). Fitted reaction rate constant $k_{[hole]}$ in different KOH concentrations (**f**).

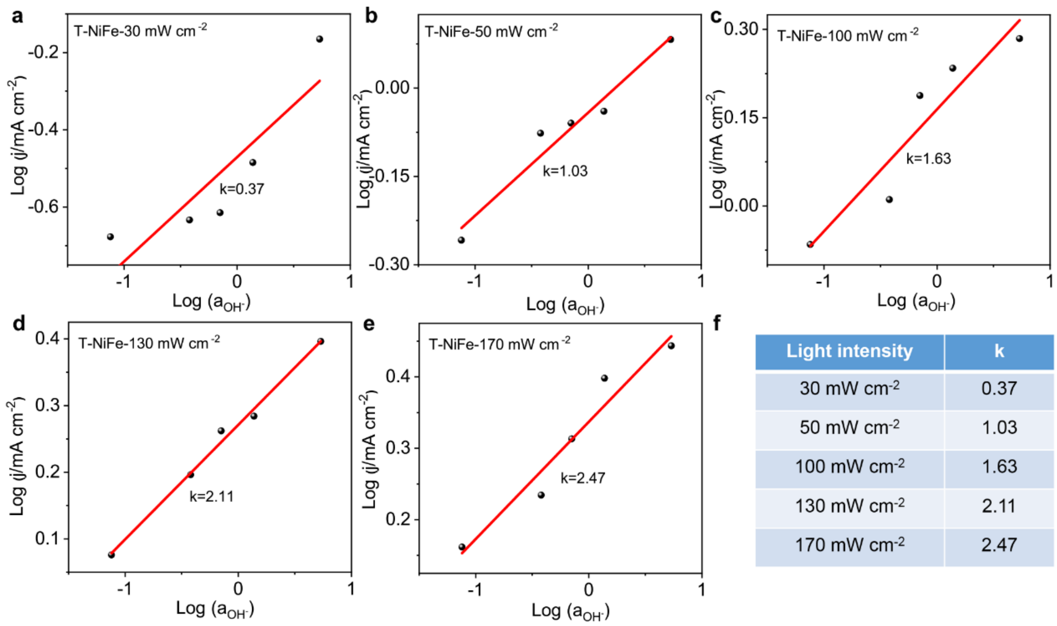

Light intensity	k
30 mW cm^{-2}	0.37
50 mW cm^{-2}	1.03
100 mW cm^{-2}	1.63
130 mW cm^{-2}	2.11
170 mW cm^{-2}	2.47

Supplementary Figure 25. Dynamic analysis. Fitted dynamic line of T-NiFe with hydroxide ion activity under 30 mW cm^{-2} (**a**), 50 mW cm^{-2} (**b**), 100 mW cm^{-2} (**c**), 130 mW cm^{-2} (**d**) and 170 mW cm^{-2} (**e**). Fitted reaction rate constant *k* under different illumination intensity (**f**).

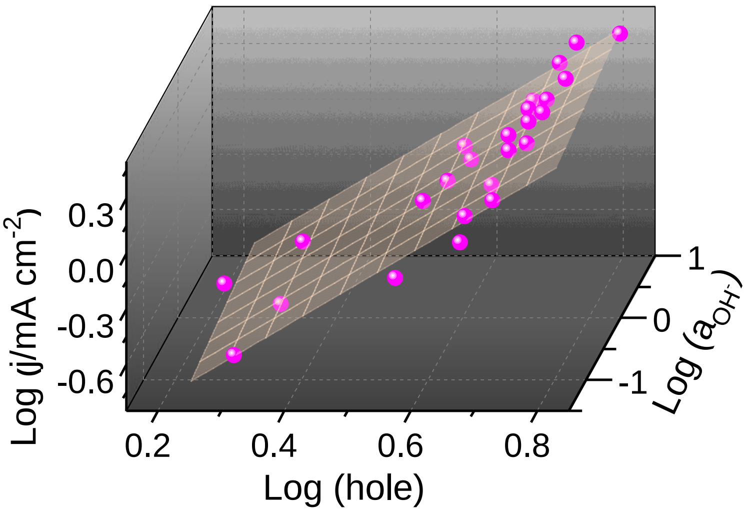

Supplementary Figure 26. Dynamic analysis. Fitted dynamic surface of T-NiFe with the function of hydroxide ion activity and surface trapped hole concentrations.

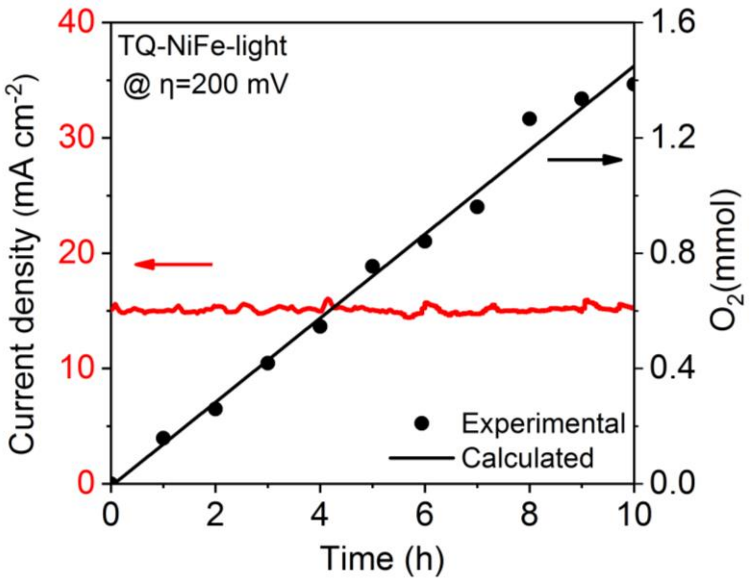

Supplementary Figure 27. Faradic efficiency. Oxygen evolution rate from gas chromatography measurement of evolved O_2 from TQ-NiFe at the overpotential of 200 mV under illumination.

The Faradic efficiency of OER refers to the conversion efficiency from electrons to oxygen molecules. The amount of evolved O_2 was monitored using a gas chromatograph. The calculation of Faradic efficiency (FE) is illustrated as follows:

$$\mathrm{FE} = \frac{4Fn_{O_2}}{It} \times 100\%$$

F is the Faraday's constant (96485 C mol^{-1}), n_{O2} is the amount of produced molecular oxygen, I is the constant current applied, and t is the reaction time.

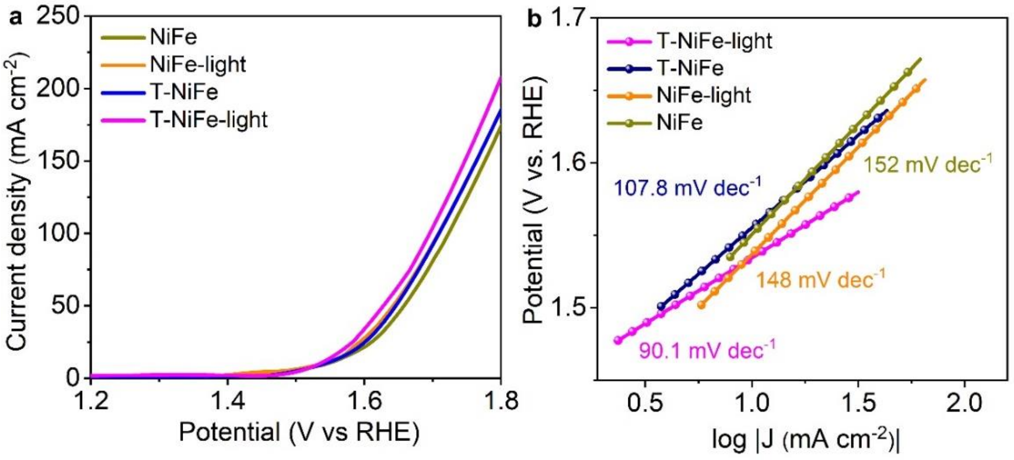

Supplementary Figure 28. Electrochemical test. OER polarization curves of T-NiFe and NiFe under and without illumination (**a**). Tafel plots of T-NiFe and NiFe under and without illumination (**b**).

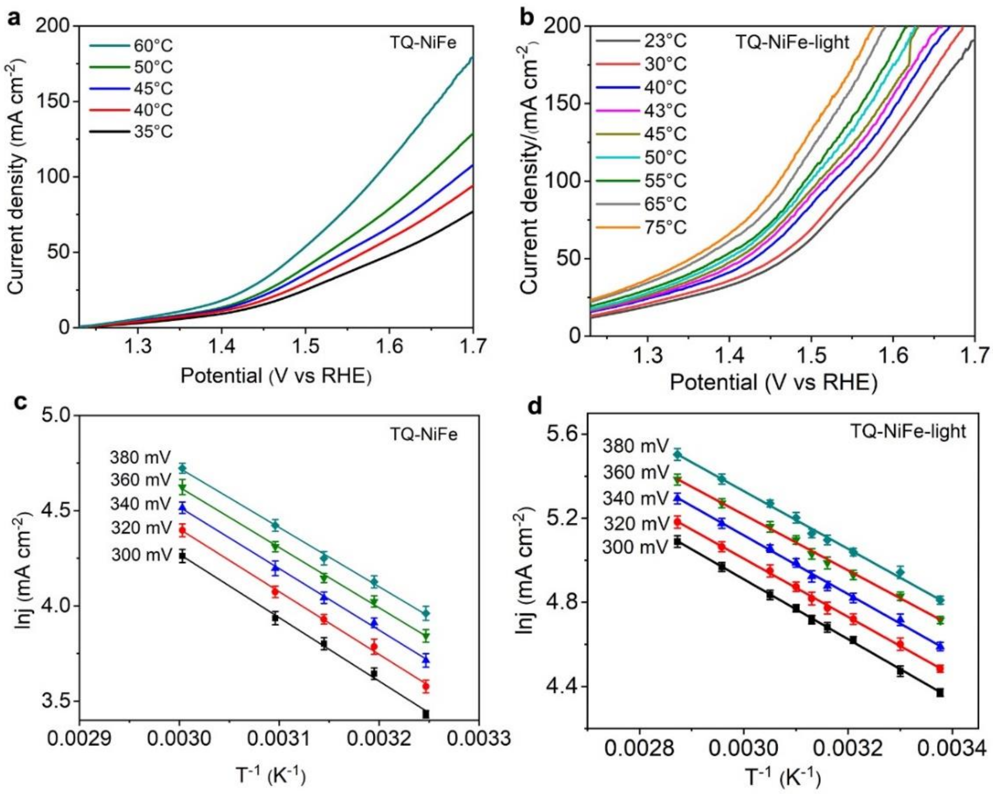

Supplementary Figure 29. Electrochemical test at different temperatures. Polarization curves at different temperatures without iR-corrected of TQ-NiFe under or without illumination (**a** and **b**). Arrhenius plots of TQ-NiFe under or without illumination (**c** and **d**). Semilogarithmic dependence of current density of at various overpotentials plotted against inverse temperature. Overpotentials are taken from 300 to 380 mV.

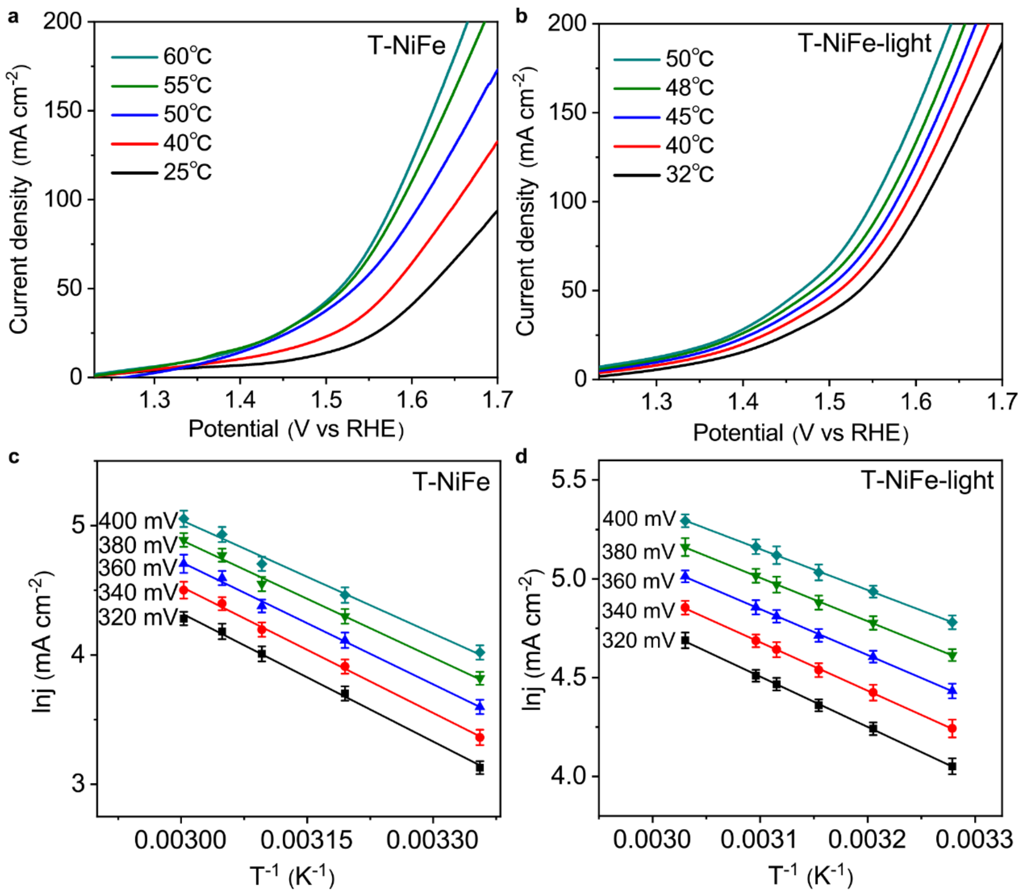

Supplementary Figure 30. Electrochemical test at different temperatures. Polarization curves at different temperatures without iR-corrected of T-NiFe under or without illumination (**a** and **b**). Arrhenius plots of T-NiFe under or without illumination (**c** and **d**): semilogarithmic dependence of current density of at various overpotentials plotted against inverse temperature. Overpotentials are taken from 320 to 400 mV.

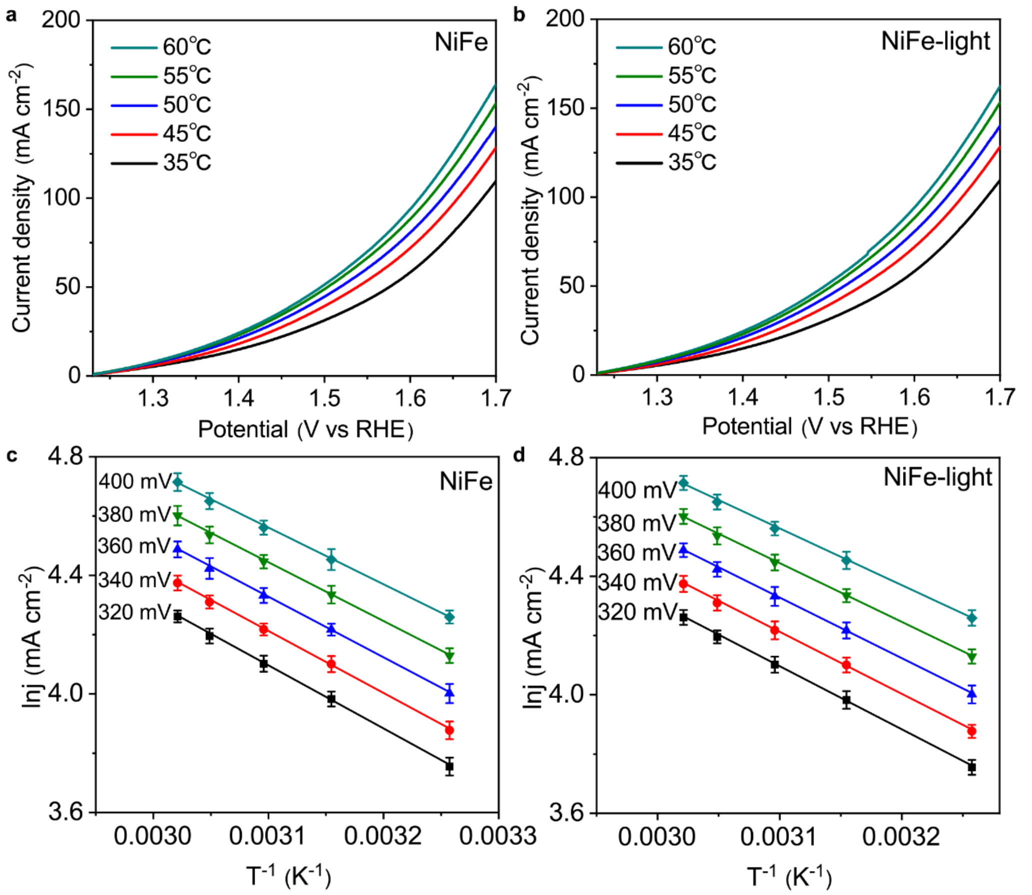

Supplementary Figure 31. Electrochemical test at different temperatures. Polarization curves at different temperatures without iR-corrected of NiFe under or without illumination (**a** and **b**). Arrhenius plots of NiFe under or without illumination (**c** and **d**): semilogarithmic dependence of current density of at various overpotentials plotted against inverse temperature. Overpotentials are taken from 320 to 400 mV.

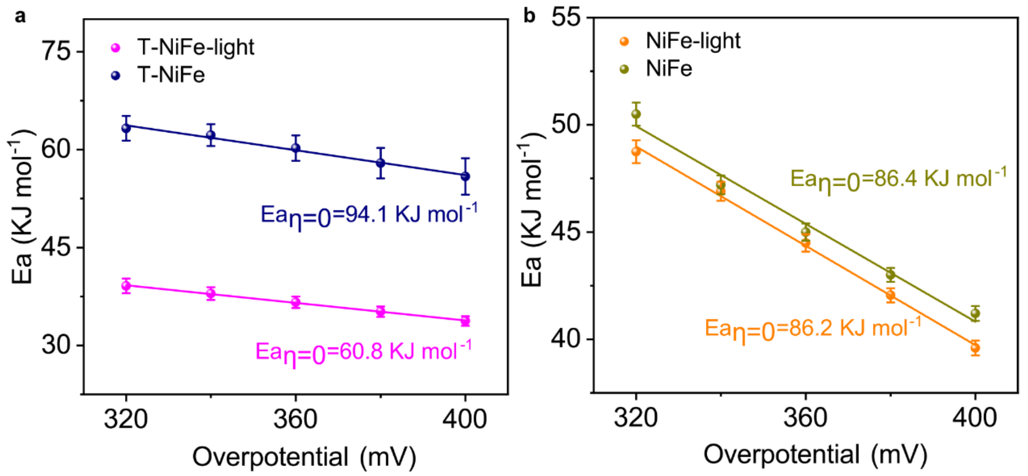

Supplementary Figure 32. Activation energy analysis. Activation energy of T-NiFe at the zero overpotential obtained through trend extrapolation with and without illumination (**a**). Activation energy of NiFe at the zero overpotential obtained through trend extrapolation with and without illumination (**b**).

Polarization curves at different temperatures for the catalysts with and without illumination were collected to assess the apparent electrochemical activation energy (E_a) for water oxidation using the Arrhenius relationship, $\frac{\partial \log(i_0)}{\partial \frac{1}{T}} = -\frac{E_a}{2.3R}$, where i_0 is the exchange current density, T is the temperature, and R is the universal gas constant. The derived Arrhenius plots were fitted at different overpotentials and the apparent electrochemical activation energy (E_a) could be figured out from the slope of curves. The plots of calculated electrochemical activation energy (E_a) versus overpotential (η) were extended to the overpotential of 0 mV, from which the apparent electrochemical activation energies (E_a) at the OER thermodynamic equilibrium potential (1.23V vs RHE) could be extracted. Polarization curves at different temperatures and Arrhenius plots of TQ-NiFe, T-NiFe and NiFe with and without illumination were shown in Supplementary Figures 28-31, which are used for further activation energy (E_a) calculation. As shown in Supplementary Figure 31, the value of the extracted E_a for NiFe under illumination hardly changed compared to that without illumination.

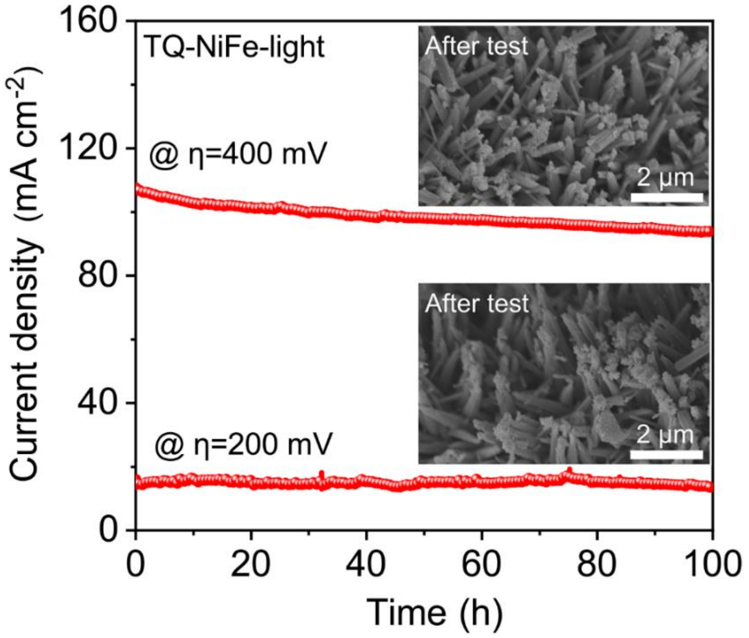

Supplementary Figure 33. Stability test. Current-time chronoamperometric (CA) curves of TQ-NiFe at the overpotential of 200 mV and 400 mV under 1.5 G illumination. The upper inset is the SEM image of TQ-NiFe after stability test at the overpotential of 400 mV. The lower inset is the SEM image of TQ-NiFe after stability test at the overpotential of 200 mV.

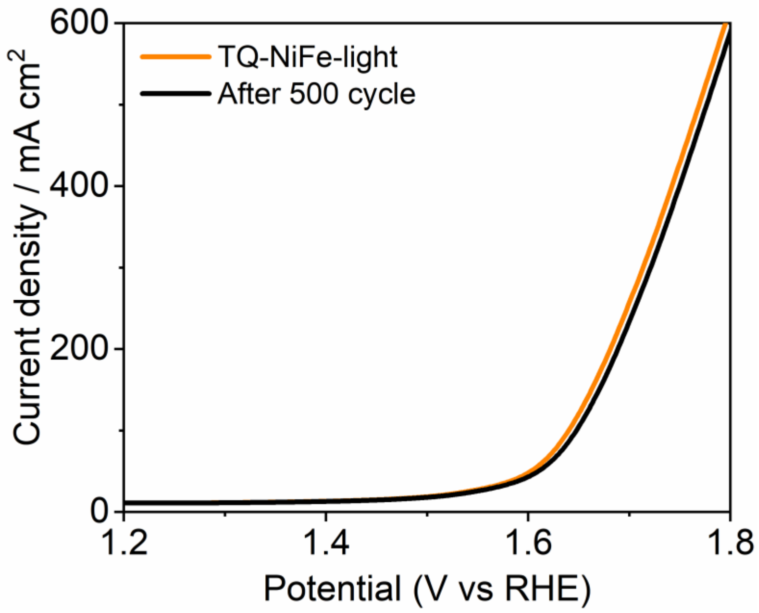

Supplementary Figure 34. Cycle performance. LSV curve of TQ-NiFe after 500 cycles under 1.5 G illumination.

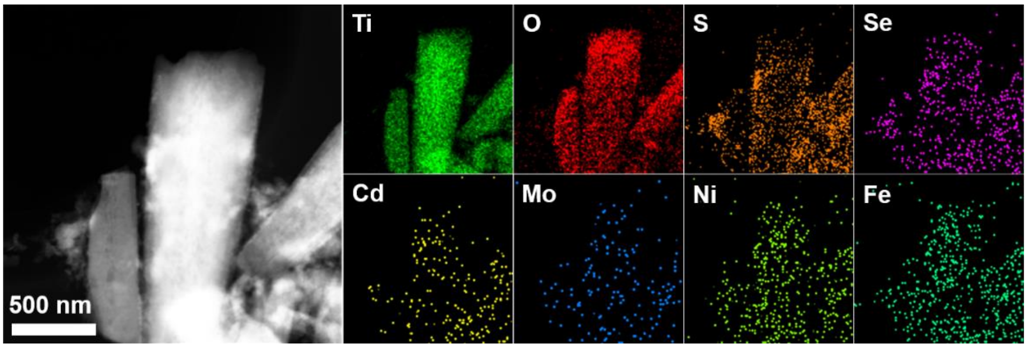

Supplementary Figure 35. HRTEM-EDS measurement. HRTEM-EDS elemental mapping images of Ti, O, Cd, S, Se, Mo, Ni and Fe in the TQ-NiFe after test.

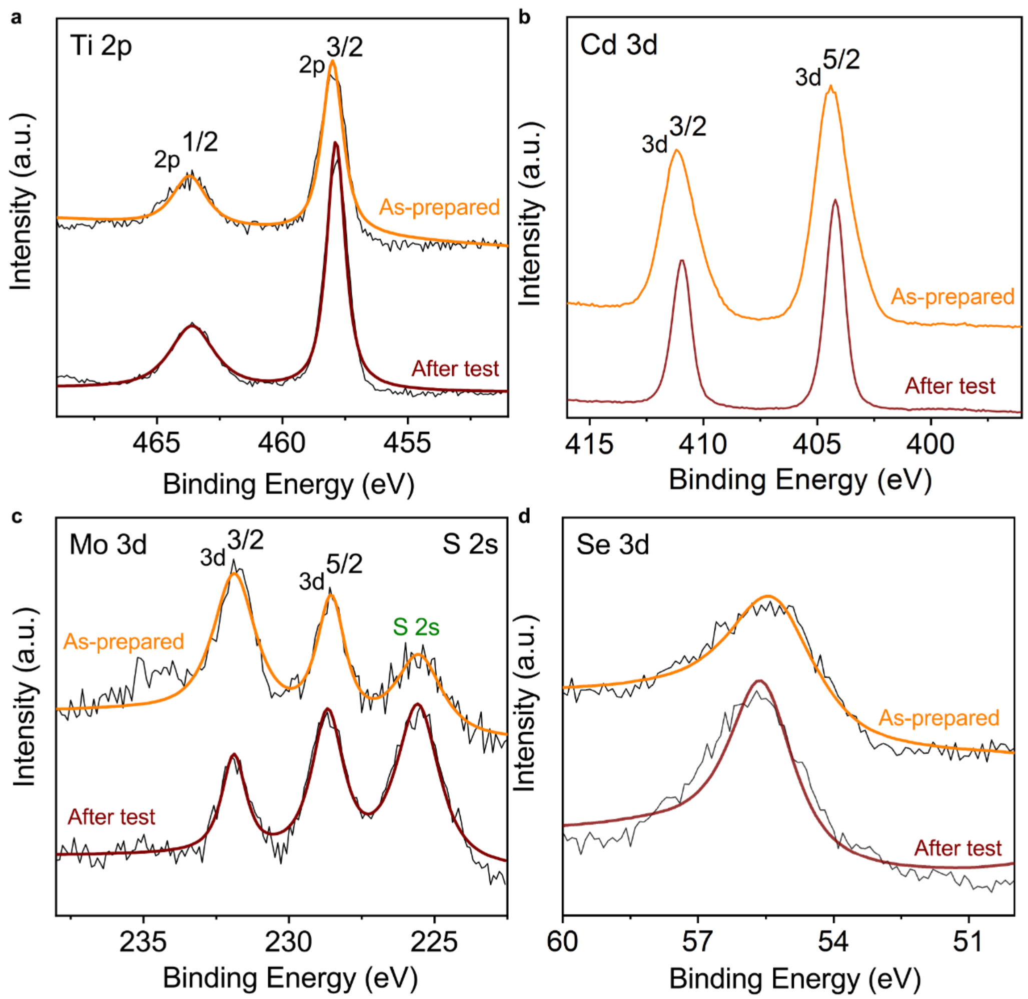

Supplementary Figure 36. X-ray photoelectron spectroscopy of TQ-NiFe after OER test. X-ray photoelectron spectroscopy of Ti 2p (**a**), Cd 3d (**b**), Mo 3d (**c**) and Se 3d **(d)** in TQ-NiFe before and after OER test.

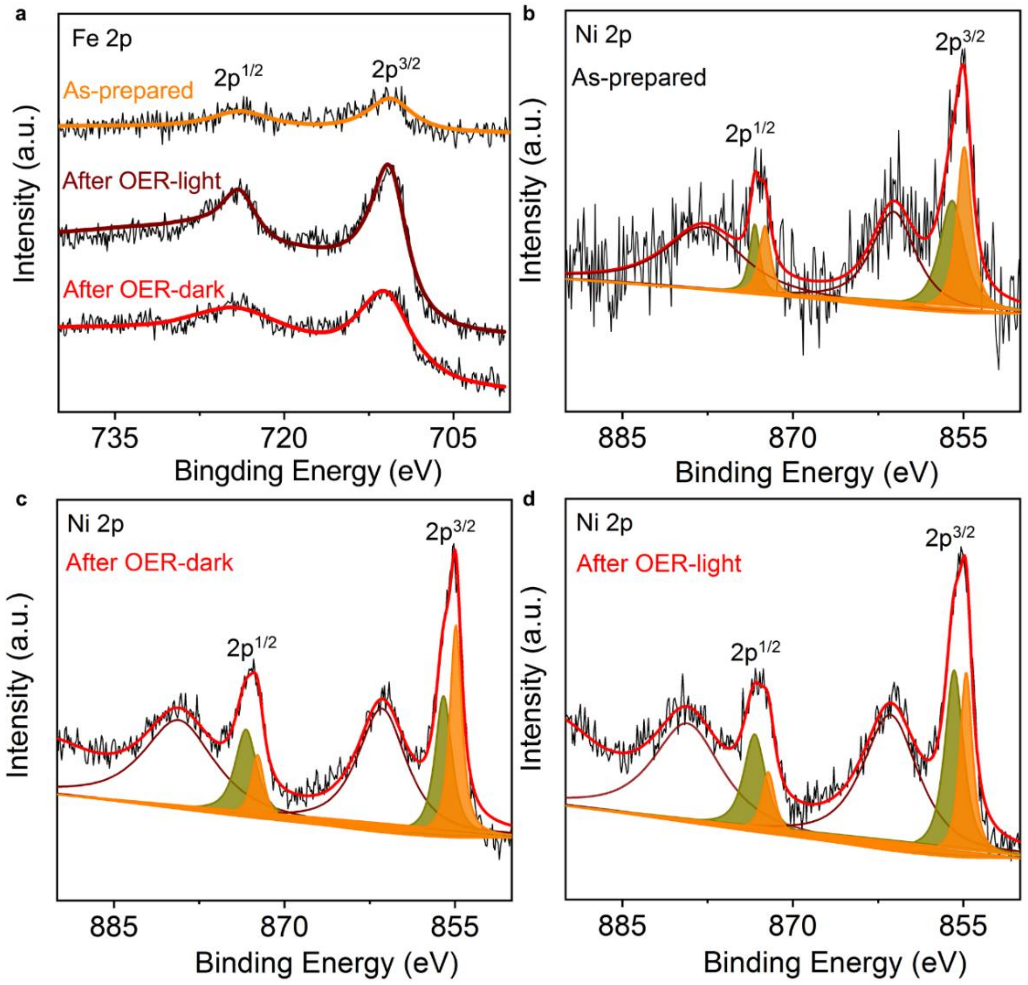

Supplementary Figure 37. X-ray photoelectron spectroscopy of TQ-NiFe after OER test. Fe 2p XPS spectra for TQ-NiFe before and after OER test (**a**). Ni 2p XPS spectra for TQ-NiFe before OER test (**b**). Ni 2p XPS spectra for TQ-NiFe after OER test without illumination (**c**) and with illumination (**d**).

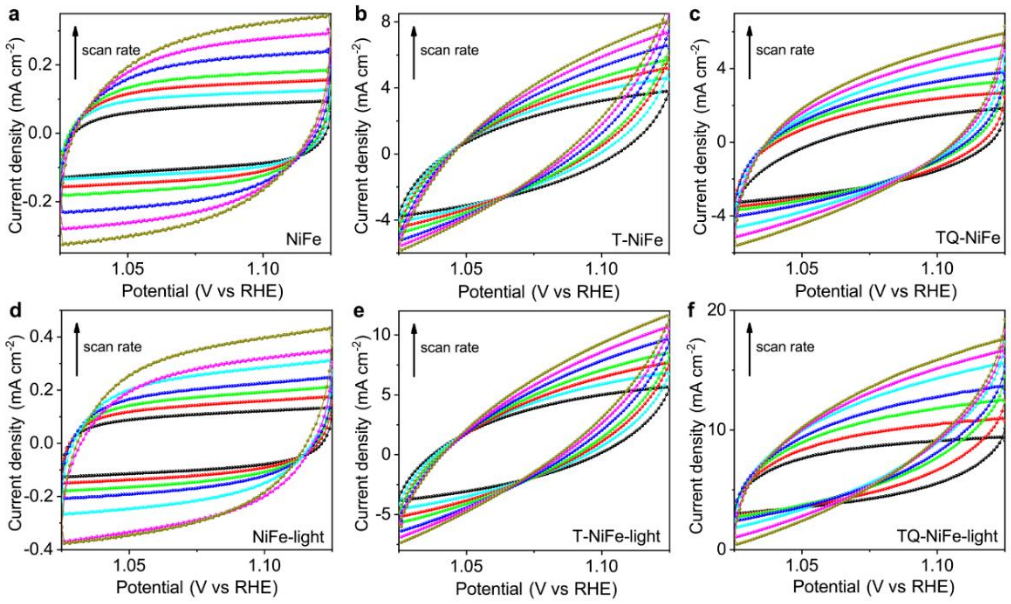

Supplementary Figure 38. CV curves under different scan rates. CV curves of TQ-NiFe (**a**), T-NiFe (**b**) and NiFe (**c**) without illumination measured at different scan rates. CV curves of TQ-NiFe (**d**), T-NiFe (**e**) and NiFe (**f**) under illumination measured at different scan rates. Scan rates are taken from 30 to 120 mV s^{-1}.

The electrochemically active surface area (ECSA) was evaluated by the double layer capacitance (C_{dl}) which was figured out from cyclic voltammetry (CV). A series of CV curves of TQ-NiFe and NiFe under or without illumination in different san rates with the potentials in the range of 1.025-1.125 V are shown in Supplementary Figure 38.

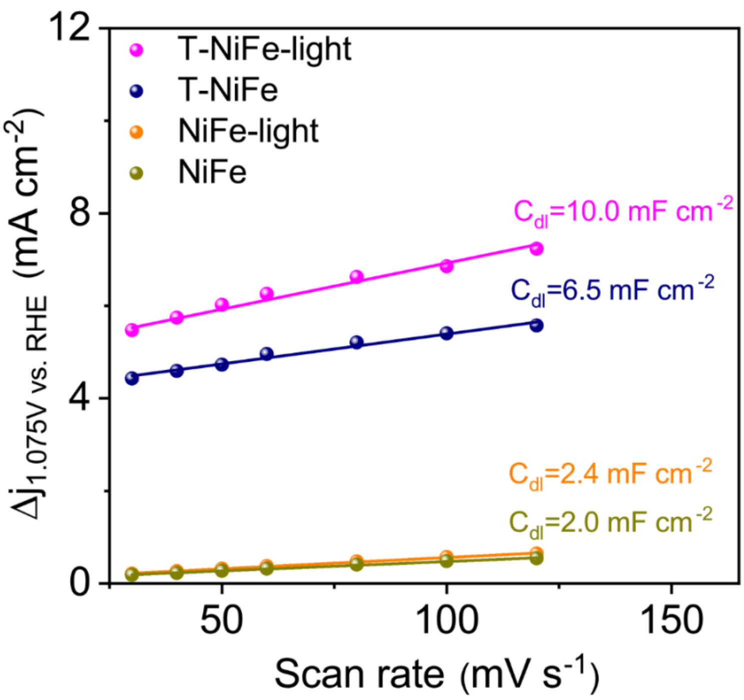

Supplementary Figure 39. Double layer capacitance. Plots of the charging current density against scan rates for T-NiFe and NiFe with and without illumination.

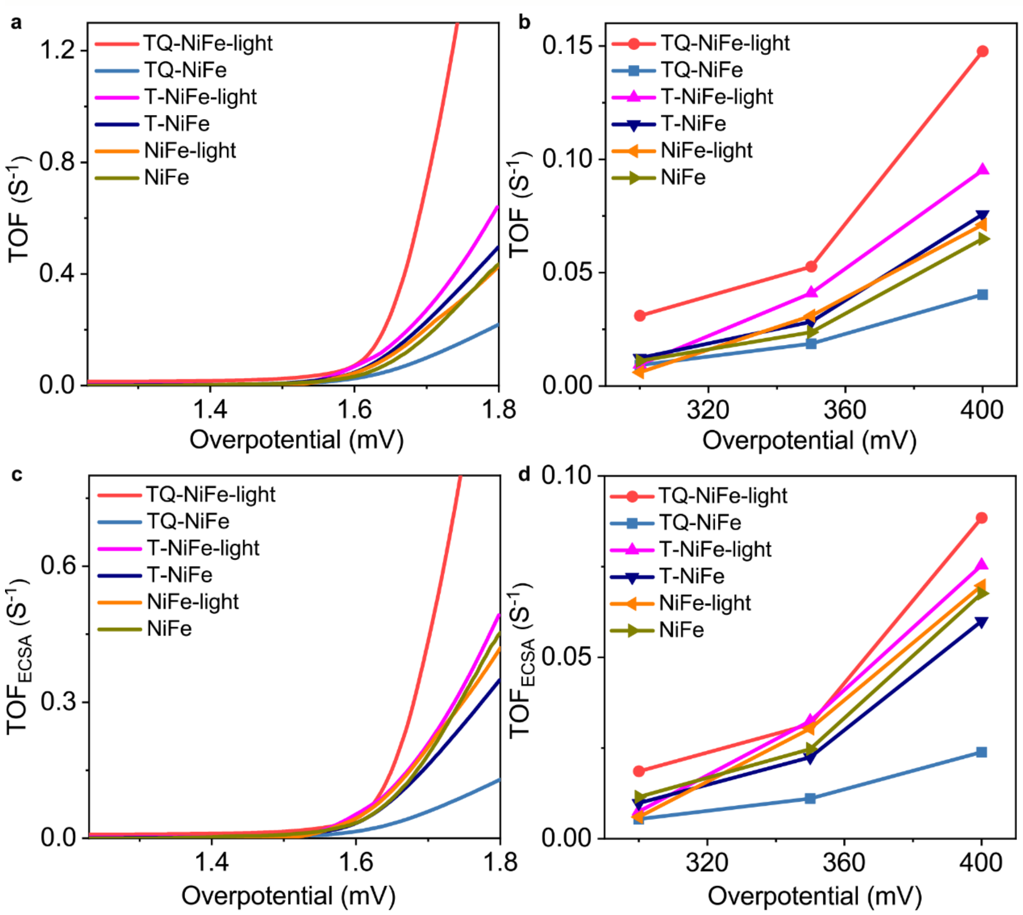

Supplementary Figure 40. Electrochemical activity assessment. TOF of TQ-NiFe, T-NiFe and NiFe under and without illumination (**a**). TOF of TQ-NiFe, T-NiFe and NiFe at the overpotential of 300, 350 and 400 mV under and without illumination (**b**). ECSA normalized TOF of TQ-NiFe, T-NiFe and NiFe under and without illumination (**c**). ECSA normalized TOF of TQ-NiFe, T-NiFe and NiFe at the overpotential of 300, 350 and 400 mV under and without illumination (**d**).

The TOF value is calculated from the equation: $TOF = \frac{J \times A}{4 \times F \times m}$, Where J is the current density at a given overpotential, A is the surface area of the electrode, F is the Faraday constant, and m is the number of moles of metal on the electrode.

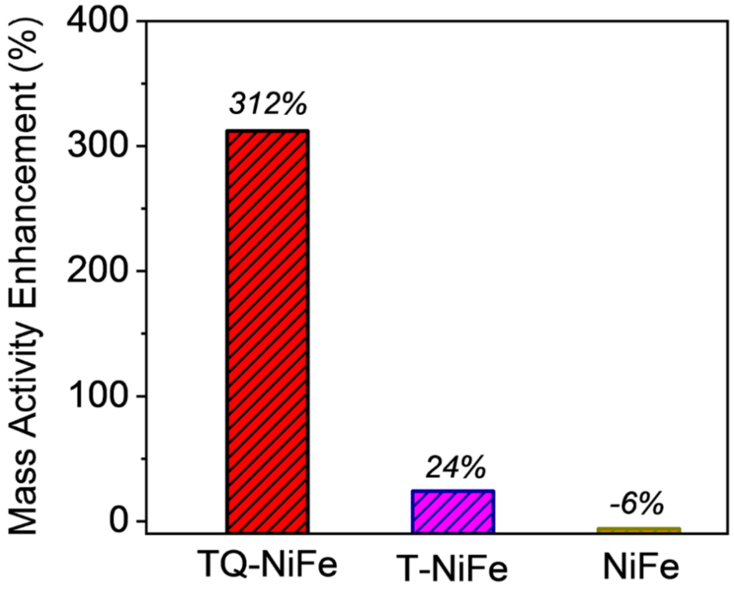

Supplementary Figure 41. Mass activity. Degree of mass activity enhancement of TQ-NiFe, T-NiFe and NiFe under 1.5 G illumination at the overpotential of 400 mV.

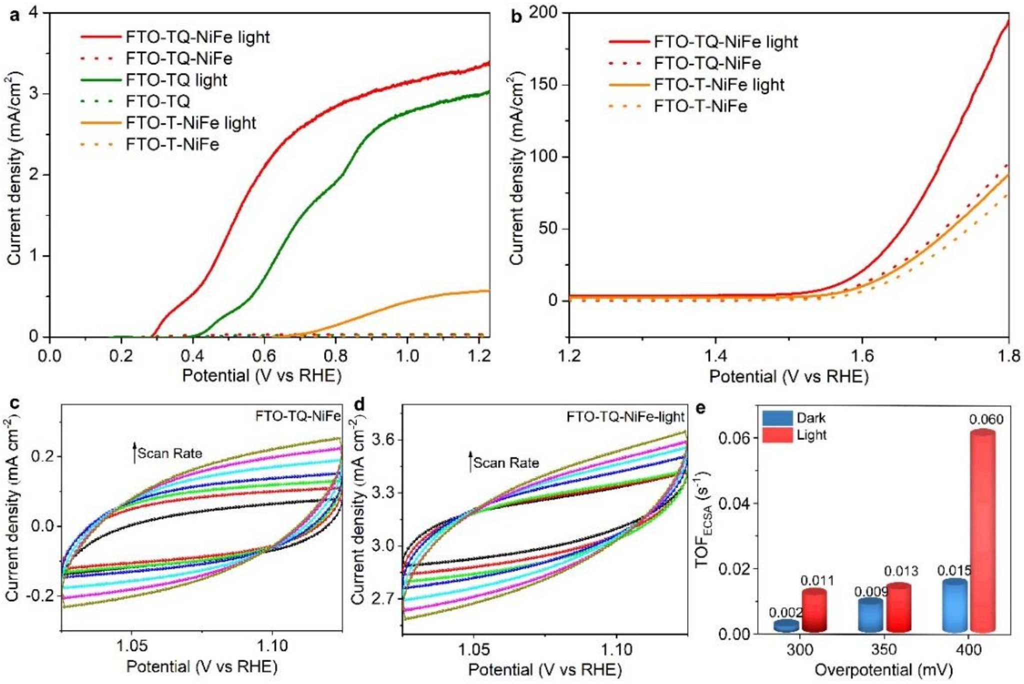

Supplementary Figure 42. PEC performance for FTO-TQ-NiFe. (**a**) PEC properties and (**b**) OER electrocatalytic properties of FTO-TQ-NiFe and FTO-T-NiFe under and without illumination. CV curves of FTO-TQ-NiFe without (**c**) and with (**d**) illumination measured at different scan rates. (**e**) ECSA normalized TOF calculated from current at different potentials.

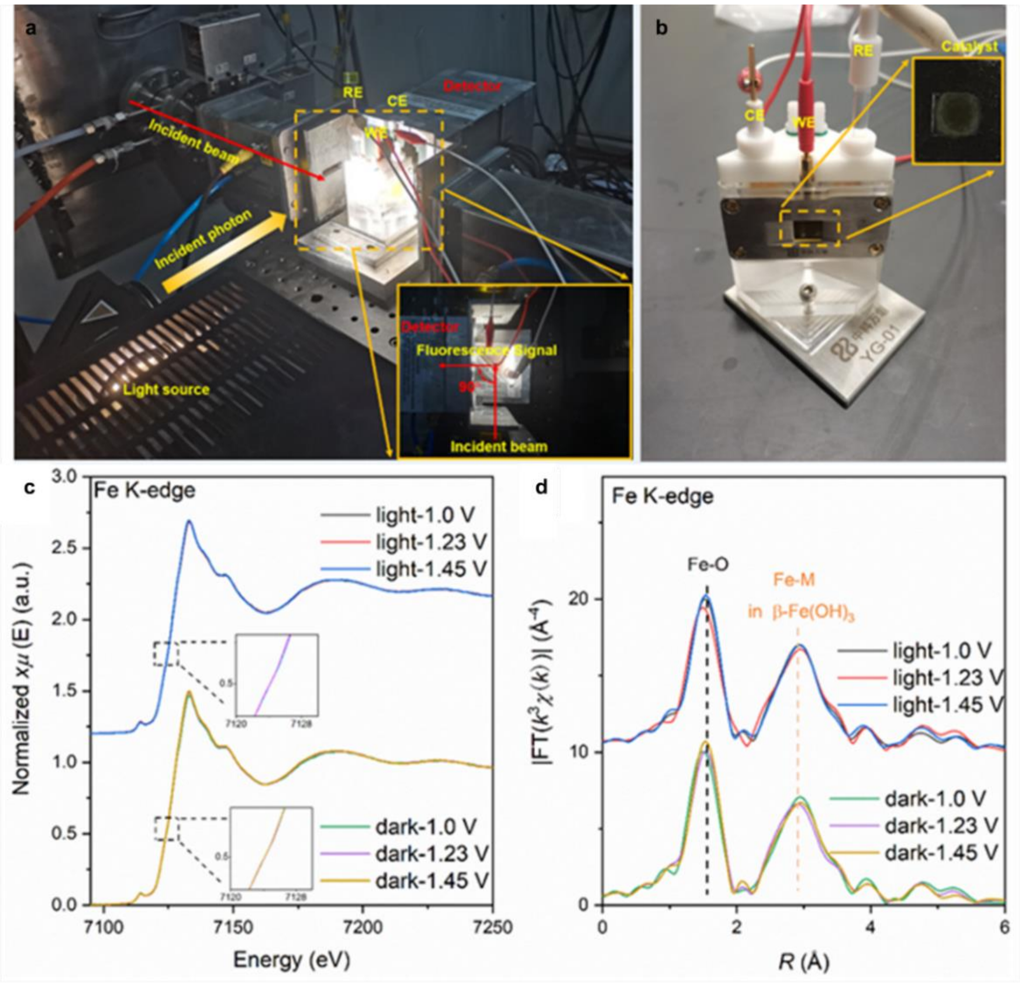

Supplementary Figure 43. XAFS analysis. Experimental setup for operando XAS measurements (a and b). A light source is used to illuminate the sample. Fe K-edge XANES spectra and Fourier-transformed k^3-weighted EXAFS signals of TQ-NiFe under 1.0, 1.23, 1.45 V without and with illumination (**c** and **d**). The inset shows the enlarged Fe K-edge XANES spectra. It can be observed that both the Fe K-edge XANES and EXAFS show almost no change during the whole OER process whether without or with illumination.

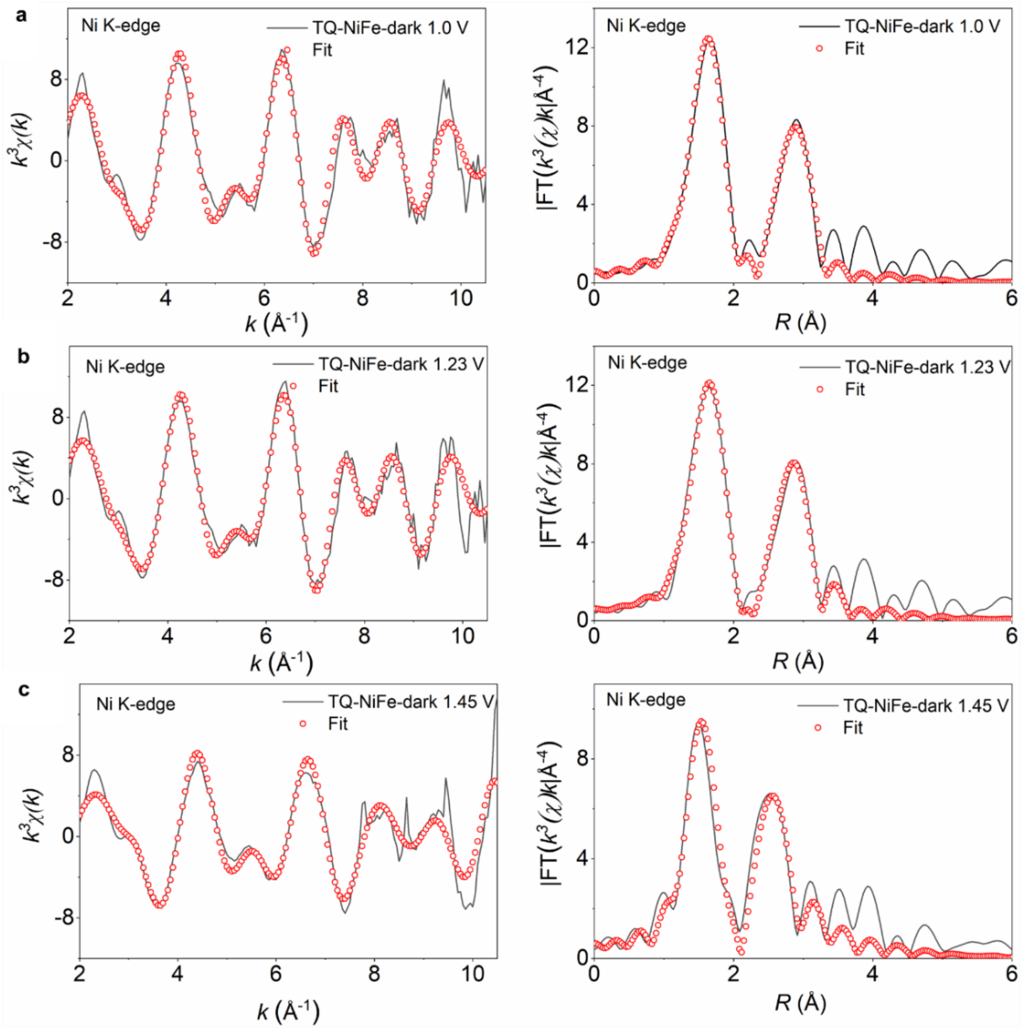

Supplementary Figure 44. Operando k^3-weighted Ni K-edge EXAFS spectra and the Fourier-transformed magnitudes for TQ-NiFe at different applied potentials without illumination. Measured and calculated spectra are matched very well. The best-fit parameters are shown in Supplementary Table 3.

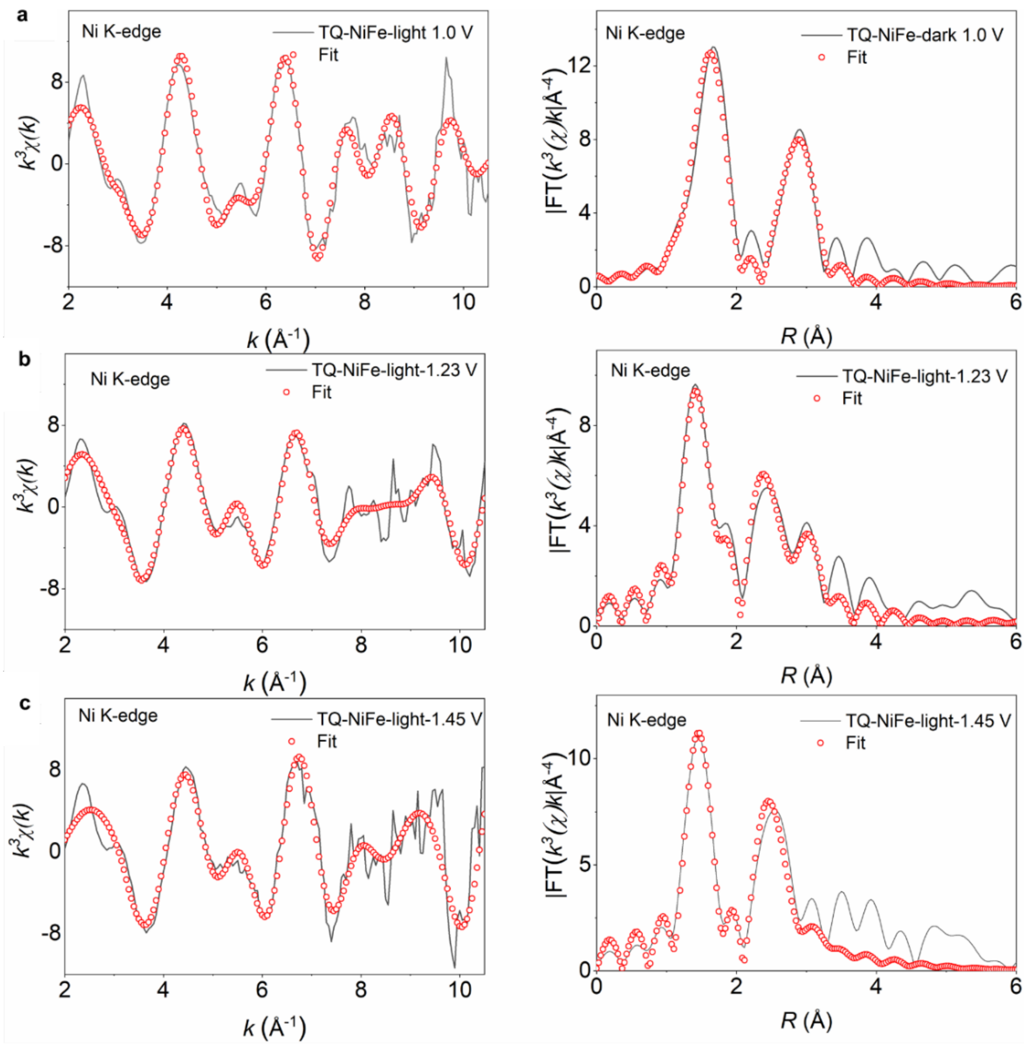

Supplementary Figure 45. Operando k^3-weighted Ni K-edge EXAFS spectra and the Fourier-transformed magnitudes for TQ-NiFe at different applied potentials with illumination. Measured and calculated spectra are matched very well. The best-fit parameters are shown in Supplementary Table 4.

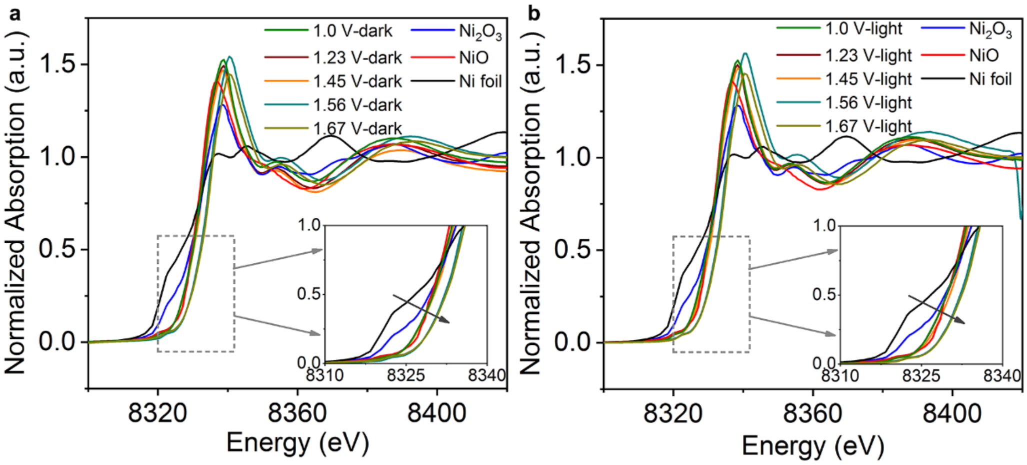

Supplementary Figure 46. XAFS analysis. Operando Ni K-edge XAS spectra of NiFe under different potentials without illumination (**a**). Operando Ni K-edge XAS spectra of NiFe under different potentials under illumination. Inset is the enlarged area from 8310 to 8340 eV (**b**).

Supplementary Table 1. Comparison of the water splitting performance for the electrocatalysts in Supplementary Figure 13.

electrocatalyst	$E_{@10\ mA\ cm-2}$ (V vs. RHE)	reference
TQ-NiFe	1.001	This work
$Cu@CeO_2@NFC_{2-x}$	1.4608	Adv. Funct. Mater. 2020, 1908367
NiFe-LDH/MXene/NF	1.459	Nano Energy 2019, 63, 103880
2D Fe-CoP/CoO	1.449	Nano Energy 2019, 56, 109
$Au/NiFeO_x$	1.42	Joule 2019, 3, 557
tannin-NiFe (TANF) complex film	1.52	Angew. Chem. Int. Ed. 2019, 58, 3769
CS-NiFeCr/Cu NA	1.43	ACS Energy Lett. 2018, 3, 2865
Ru_1-Pt_3Cu	1.45	Nature Catalysis 2019, 2, 304
Core-shell NiFeCu/NF	1.41	Nat. Commun. 2018, 9, 381
(Ni,Fe)OOH/NF	1.384	Energy Environ. Sci. 2018, 11, 2858
NiFe LDHs–V_{Ni}	1.459	Small 2018, 14, 1800136
Fe^{2+} -NiFe LDH	1.479	Angew. Chem. Int. Edit. 2018, 57, 9392
NiFeRu LDH/Ni foam	1.455	Adv. Mater. 2018, 30, 1706279
NiFe Prussian blue analogue	1.467	J. Am. Chem. Soc. 2018, 140, 11286
Au/NiFe LDH	1.496	J.Am.Chem.Soc. 2018, 140, 3876
CeO_x/CoS	1.499	Angew. Chem. Int. Edit. 2018, 57, 8654
NiFe Hydroxide Film	1.47	Adv. Energy Mater. 2017, 7, 1602547
NiFe-Pt LDH	1.46	Nano Energy 2017, 39, 30
CoFe LDH@Cu NWs	1.47	Nano Energy 2017, 41, 327
NiFe LDH NS@DG10	1.44	Adv. Mater. 2017, 29, 1700017
H_2O-plasma CoFe LDH	1.462	Adv. Mater. 2017, 29, 1701546
$NiO/Co_3O_4@NC$	1.43	ACS Energy Lett. 2017, 2, 2177
$(Ni_{0.5}Fe_{0.5})_2P$/Ni foam	1.433	J. Mater. Chem. A 2017, 5, 11229

Ni_3FeN/Co,N-CNF	1.5	Nano Energy 2017, 40, 382
NiFe-oxide nanocube	1.501	ACS Appl. Mater. Inter. 2017, 9, 41906
spinel-type $Zn_xCo_{3-x}O_4$ films	1.56	ACS Appl. Mater. Inter. 2017, 9, 17186
α-$Co_4Fe(OH)_x$	1.525	J. Mater. Chem. A 2017, 5, 1078
NiFe@NC	1.488	Nano Energy 2017, 39, 245
NiFe LDH@Cu NWs	1.429	Energy Environ. Sci. 2017, 10, 1820
NiCoFe LDHs/CFC	1.469	ACS Energy Lett. 2016, 1, 445
3D Ir	1.47	Nano Lett. 2016, 16, 4424
gelled FeCoW oxyhydroxides	1.453	Science 2016, 352, 333
$Ni_xFe_{1-x}Se_2$-DO	1.425	Nat. Commun. 2016, 7, 12324
CoAl-NS	1.482	Adv. Mater. 2016, 28, 7640.
NiFe LDH HMS	1.469	ACS Appl. Mater. Inter. 2016, 8, 3697
Co_4N nanowire	1.487	Angew. Chem. 2015, 127, 14923
CoO-MoO_2/NF	1.5	Nanoscale 2015, 7, 16704

Supplementary Table 2. Comparison of the water splitting performance for the photoelectrocatalysts in Supplementary Figure 13.

photoelectrocatalyst	$J_{@1.23\ V\ vs\ RHE}$ (mA cm^{-2})	reference
TQ-NiFe	10.74	This work
$CdS/ZnFe_2O_4/Cu_2O$	3.3	Appl. Catal. B-Environ., 2020, 268, 118460
FTO IOs/CdS NRs/CdSe	9.2	Small, 2020, 16, 1905826
FeOOH/V-NiOOH/BVO	5.43	Angewandte Chemie, 2020, 59, 6213-6218
WO_3/CdS/NiOOH/Co-Pi	2.8	J. Alloy. Compd. 2019, 790, 493-501
H-TiO_2	2.5	Adv. Energy Mater. 2019,9, 1900725
Al-ZnO/CdS/TiO_2	11.7	Appl. Catal. B-Environ., 2019, 255, 117738
TiO_2/CdS/MoS_2	3.25	Appl. Catal. B-Environ., 2019, 259, 118102
ZnO/CdS/PbS	14.2	Chem. Eng. J., 2019, 362, 658-666
$TiO_2/BiVO_4$	3.3	RSC Adv., 2018, 8, 41439-41444
CdS/SnS_x	1.59	Adv. Funct. Mater., 2018, 28, 1706785
mp-CdS/TiO_2	7.8	ACS Appl. Mater. Interfaces 2018, 10, 23766-23773
Al-ZnO/CdS	10.4	Nanoscale, 2018, 10, 19621-19627
CdS NR@SnO_2	3.8	Small, 2018, 14, 1801352
Au NPs/CdS/ZnO	15	ACS Sustain. Chem. Eng. 2017, 5, 4249
Fe_2O_3/CdS	2.32	Appl. Catal. B-Environ., 2017, 218, 570-580
CdS@$CdIn_2S_4$	5.5	Nanoscale, 2017,9, 6296-6301
CdS-MoS_2	4	RSC Adv., 2017, 7, 44626-44631
a-Si:H/anodized Ni-Fe	4.6	Adv. Energy Mater. 2017, 1700659
ZFO/CdS/ZnO	3.88	Nano Energy, 2016, 24, 25-31
CdS/CdSe-SnO_2/TiO_2	6	RSC Adv., 2016, 6, 37407-37411
CdS/WO_3	1.8	RSC Adv., 2016, 6, 16668-16672

CdS QD-SNWs @ SNSs	9.9	Nano Energy, 2016, 19, 318-327
Ti-Fe_2O_3/CdS	2.7	RSC Adv., 2016,6, 74234-74240
TiO_2/CdS/Co-Pi	1.05	Adv. Funct. Mater. 2015, 25, 5706-5713
CdS QDs-$BaSnO_3$ NWs	4.8	J. Mater. Chem. A 2015, 3, 12769-12776
np^+-Si/TiO_2/$NiCrO_x$	3.8	Energy Environ. Sci., 2015, 8, 203-207
Co-Pi^+Co$(OH)_x$/NiFe-LDH/Ta_3N_5	6.3	Chem. Mater. 2015, 27, 2360-2366
Sn,Zr-Fe_2O_3-NiOOH	1.65	J. Mater. Chem. A, 2015, 3, 5949-5961.

Supplementary Table 3. Ni *K*-edge EXAFS curve Fitting Parameters of TQ-NiFe-dark at selected potentials[a]

sample	path	N	R (Å)	σ^2 ($\times10^{-3}$ Å^2)	ΔE_0 (eV)	R, %
1.0 V[b]	Ni-O1<br>Ni-M1<br>Ni-O2	6.0<br>4.5<br>5.0	2.04<br>3.05<br>3.40	9.1<br>6.0<br>3.0	5.2	0.2
1.23 V[b]	Ni-O1<br>Ni-M1<br>Ni-O2	6.0<br>4.8<br>5.0	2.04<br>3.05<br>3.40	9.7<br>6.3<br>6.6	5.3	0.2
1.45 V[b]	Ni-O1<br>Ni-M1<br>Ni-O2	5.1<br>3.4<br>4.8	1.98<br>2.89<br>3.43	8.0<br>4.0<br>3.0	7.3	0.3

[a]N, coordination number; R, distance between absorber and backscatter atoms; σ^2, Debye-Waller factor to account for both thermal and structural disorders; ΔE_0, inner potential correction; R factor (%) indicates the goodness of the fit. Error bounds (accuracies) that characterize the structural parameters obtained by EXAFS spectroscopy were estimated as $N \pm 20\%$; $R \pm 1\%$; $\sigma^2 \pm 20\%$; $\Delta E_0 \pm 20\%$. S_0^2 was fixed to 1.0. Bold numbers indicate fixed coordination number (N) according to the crystal structure. [b]Fitting range: $2.0 \leqslant k$ (/Å) $\leqslant 10.5$ and $1.0 \leqslant R$ (Å) $\leqslant 3.3$.

Supplementary Table 4. Ni *K*-edge EXAFS curve Fitting Parameters of TQ-NiFe-light at selected potentials[a]

sample	path	*N*	*R* (Å)	σ^2 ($\times 10^{-3}$ Å^2)	ΔE_0 (eV)	*R*, %
1.0 V[b]	Ni-O1<br>Ni-M1<br>Ni-O2	6.0<br>4.0<br>6.0	2.03<br>3.05<br>3.40	8.5<br>5.0<br>3.0	5.7	0.2
1.23 V[b]	Ni-O1<br>Ni-O2<br>Ni-M1<br>Ni-M2	2.4<br>2.3<br>2.6<br>3.0	1.94<br>2.17<br>2.83<br>3.01	3.5<br>4.4<br>14.3<br>3.6	9.3	0.3
1.45 V[b]	Ni-O1<br>Ni-O2<br>Ni-M1<br>Ni-M2	3.7<br>1.3<br>2.8<br>2.3	1.91<br>2.15<br>2.86<br>3.03	3.5<br>3.0<br>13.7<br>4.8	9.7	0.3

[a]*N*, coordination number; *R*, distance between absorber and backscatter atoms; σ^2, Debye-Waller factor to account for both thermal and structural disorders; ΔE_0, inner potential correction; *R* factor (%) indicates the goodness of the fit. Error bounds (accuracies) that characterize the structural parameters obtained by EXAFS spectroscopy were estimated as $N \pm 20\%$; $R \pm 1\%$; $\sigma^2 \pm 20\%$; $\Delta E_0 \pm 20\%$. S_0^2 was fixed to 1.0. Bold numbers indicate fixed coordination number (*N*) according to the crystal structure. [b]Fitting range: $2.0 \leqslant k$ (/Å) $\leqslant 10.5$ and $1.0 \leqslant R$ (Å) $\leqslant 3.3$.

References:

1. Fabre E., Mautref M., Mircea A., Trap saturation in silicon solar cells, *Appt. Phys. Lett.,* **27**, 239 (1975)

2. Ho C. F., Bell R. O., Wald F. V., Enhancement of diffusion length in EFG ribbon solar cells under illumination, *Appl. Phys. Lett.,* **31**, 463 (1977).

3. Pandya R. Schiff E. A., A multiple-trapping model with optical bias, *Philosophical Magazine B,* **52**, 1075-1095 (1985).

4. Kroeze J. E., Savenije T. J., Warman J. M., Electrodeless determination of the trap density, decay kinetics, and charge separation efficiency of dye-sensitized nanocrystalline TiO_2, *J. Am. Chem. Soc.,* **126**, 7608-7618 (2004).

5. Jennings J. R., Ghicov A., Peter L. Schmuki M., P., Walker A. B., Dye-sensitized solar cells based on oriented TiO_2 nanotube arrays: transport, trapping, and transfer of electrons, *J. Am. Chem. Soc.,* **130**, 13364-13372 (2008).

6. Schwarzburg K., Willig F., Influence of trap filling on photocurrent transients in polycrystalline TiO_2, *Appl. Phys. Lett.,* **58**, 2520 (1991).

7. Katz O., Bahir G., Salzman J., Persistent photocurrent and surface trapping in GaN Schottky ultraviolet detectors, *Appl. Phys.Lett.,* **84**, 4092 (2004).

8. AlOtaibi B., Nguyen H. P. T., Zhao S., Kibria M. G., Fan S., Mi Z., Highly stable photoelectrochemical water splitting and hydrogen generation using a double-band InGaN/GaN core/shell nanowire photoanode, *Nano Lett.,* **13**, 4356-4361 (2013).

9. Thomas J. M., Fuding L., Boettcher S. W., Theory and simulations of electrocatalyst-coated semiconductor electrodes for solar water splitting, *Phys. Rev. Lett.,* **112**, 148304 (2014).
